# A Novel Coprecipitation Path to a High‐Performing Ni/MgO Catalyst for Carbon Dioxide Methanation

**DOI:** 10.1002/cssc.202502052

**Published:** 2025-10-09

**Authors:** Anna Wolf, Michael Chumakovski, Hauke Rohr, Patrik Hauberg, Morteza Saedi, Sebastian Mangelsen, Malte Behrens

**Affiliations:** ^1^ Institute of Inorganic Chemistry Kiel University Max‐Eyth‐Str. 2 Kiel 24118 Germany

**Keywords:** coprecipitation, methanation, nanoparticles, nickel catalysts, solid solution

## Abstract

The novel crystalline bimetallic single‐source precursor (Ni_1–*x*_Mg_*x*_)_12_(CO_3_)_8_(OH)_6_O · *y* H_2_O with *x* = 0–0.5 can be converted into a highly active Ni/MgO CO_2_ methanation catalyst. All stages of preparation, namely, coprecipitation, crystallization, calcination, and reduction, as well as the spent catalysts have been comprehensively analyzed using powder X‐ray diffraction, physisorption, transmission electron microscopy, and other techniques. The scalable synthesis allows attaining unusually high surface areas around 230 m^2^ g^−1^ for the calcined precatalyst Ni_1–*x*_Mg_*x*_O. During reduction, this oxide solid solution separates into metallic Ni and Ni‐depleted oxide to form the active catalyst with finely interdispersed nanoparticles of both components with a high porosity. A high methane production rate is observed in a CO_2_/H_2_ (1:4) feed at high space velocities of ≈150 Lh^−1^ g^−1^. This performance is competitive with an industrial methanation catalyst and depends strongly on the Ni:Mg ratio utilized in the synthesis. For an equimolar ratio, the new catalyst is found to be 4 times as active as the benchmark. Due to the nanoscaled microstructure, the novel material can stabilize very high Ni loadings (≤77 wt%) with only minor sintering effects at a reaction temperature of 240–280 °C. This material thus closes the gap between thermally unstable Raney‐type and conventional lower loaded impregnated industrial catalysts.

## Graphical abstract

A highly active Ni/MgO CO_2_ methanation catalyst is derived from a novel crystalline bimetallic single‐source precursor. This material is characterized by its high specific surface area and interdispersed nanoparticles originating from a solid solution.

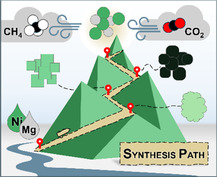

## 1 Introduction

The demand for chemical storage of renewable energy is a current challenge in the context of the transformation of the energy sector toward sustainability. There are already various facilities operating at different levels of the “power‐to‐gas” (PtG) concept converting anthropogenic CO_2_ with so‐called green hydrogen to methane^[^ ^1^, ^2^, ^3^ ^]^ while economic and political aspects are still under a delaying debate.^[^ ^4^, ^5^ ^]^ Development of new catalytic materials with superior performance in such reactions holds great potential to increase efficiency of the underlying processes and to accelerate their implementation highlighting the critical role of synthetic inorganic chemistry of new catalysts and of the relevant solid‐state chemistry.

For the methanation reaction of carbon dioxide (Sabatier reaction, Equation (1)), Ni catalysts are often applied due to their high activity and low cost compared to noble metals. They have been studied in numerous combinations with different supporting oxides to achieve high specific surface areas of Ni nanoparticles.^[^ ^3^ ^]^

(1)

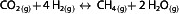




Magnesium oxide has been studied as support and promoter for nickel catalysts in various reactions alike. It has been found that MgO inhibits the disproportionation of CO within the Boudouard equilibrium in these reactions and therefore tends to make the catalyst resistant to coking.^[^ ^4^, ^6^, ^7^, ^8^, ^9^, ^10^ ^]^ A current review^[^ ^11^ ^]^ summarizes the status of research on selected catalysts originating from NiO–MgO solid solution precatalysts for various application areas such as dry methane reforming, CO_2_ hydrogenation, partial methane oxidation, and steam reforming of hydrocarbons. A special feature of catalysts obtained from a solid solution is the prevention of sintering and coking.^[^ ^4^, ^9^, ^10^, ^11^ ^]^ On the other hand, disadvantages are that often high temperatures are required to generate the solid solution, which might lead to low porosity and low specific surface areas limiting the amounts of Ni that can be stabilized. Consequently, for base metal catalysts relatively low loadings (<30%) are used in most of the solid solution precatalysts previously presented. Bulk catalysts with higher porosity and larger Ni surface areas are Raney‐type or skeletal catalysts, which, however, lack thermal stability due to absence of a support and are applied only in low‐temperature applications. Typical impregnated catalysts, on the other hand, may lack the abovementioned advantages of the solid solution precatalyst. In addition, MgO that can be used for impregnation experiments is not as easy to obtain as a large specific surface area material like the more typical alumina or silica supports (>100 m^2^ g^−1^).

To explore the gap between unstable Raney Ni and low‐loaded impregnated catalysts and to exploit the promising potential of solid solution precatalysts, a Ni/MgO catalyst with a high dispersion and large surface area, high loading, and high stability seems desirable, which requires a nanostructured precursor for the solid solution that can be transformed at relatively low temperature yielding a solid solution with high surface area. A proven approach toward such goal is the use of a coprecipitated crystalline single‐source precursor, which contains already all required metals in the targeted ratio and allows to be converted first into the solid solution precatalyst and finally the active catalyst by mild thermal post‐treatment, i.e., calcination and reduction. Ideally, a large fraction of interfaces between the support and metal catalyst is generated under retention of a large surface area and a small crystallite size (cs).

An archetypical example is the industrial synthesis of the Cu/ZnO resp. Cu/ZnO/Al_2_O_3_ methanol synthesis catalyst,^[^ ^12^, ^13^, ^14^ ^]^ for which the coprecipitated precursor is zincian malachite, a hydroxy carbonate mineral which contains Cu as well as Zn in a joint cationic sublattice.^[^ ^12^, ^15^ ^]^ Despite the success and the long history of this specific catalyst synthesis, it largely remained a peculiarity in industrial catalyst manufacture and has not been transferred systematically to other elements and new base‐metal catalysts. For nickel, the use of such hydroxy carbonate structure as a precursor for a heterogeneous catalyst is indeed new since nickel often favors to crystallize in a hydroxide or hydrotalcite material when precipitated.^[^ ^16^ ^]^

An obvious candidate for a precursor would be Ni_2_(CO_3_)(OH)_2_, which occurs as mineral named nullaginite and is structurally similar to malachite. However, reports about its synthesis and substitution chemistry are sparse^[^ ^17^, ^18^ ^]^ and no synthetic crystalline material has been reported to the best of our knowledge. Other candidate materials for precursors might be substituted version of the carbonates of nickel, such as Ni(CO_3_) · 5.5H_2_O (hellyerite),^[^ ^19^ ^]^ or the anhydrous variant gaspéite (NiCO_3_). In addition to other variants,^[^ ^19^ ^]^ a synthetic nickel hydroxycarbonate (Ni_12_(CO_3_)_8_(OH)_6_O · *y* H_2_O)^[^ ^20^ ^]^ without an equivalent mineral is also known since a decade. Since Ni and Mg have very similar ionic radii, a substitution is probable and to be expected for this compound as it has already been proven in the case of layered double hydroxides and oxides.^[^ ^11^, ^16^ ^]^

**Figure 1:**
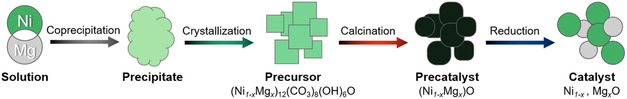
Path of synthesis for a Ni/MgO catalyst derived from the novel coprecipitated (Ni_1–*x*_Mg_*x*_)_12_(CO_3_)_8_(OH)_6_O · *y* H_2_O precursor.

Herein, we present novel Ni/MgO catalysts for methanation of CO_2_ derived from this single‐source precursor that fulfils the abovementioned requirements and closely resembles the elegant and successful synthesis of industrial Cu/ZnO catalyst, thus expanding its synthetic concept to new base‐metal catalysts. Starting point of our study is the development of the synthetic route for (Ni_1–*x*_Mg_*x*_)_12_(CO_3_)_8_(OH)_6_O · *y* H_2_O with *x* = 0–0.5 by coprecipitation followed by hydrothermal ageing. Subsequently, the precatalyst (Ni_1–*x*_Mg_*x*_O solid solution) is prepared by calcination and subsequently subjected to reduction to form the active catalyst. The process is shown schematically in **Figure** **1**.

The steps are analytically corroborated by an extensive set of complementary characterization methods, including chemical and structural analysis by inductively couples plasma optical emission spectroscopy (ICP‐OES), enery‐dispersive X‐ray spectroscopy (EDX), and powder X‐ray diffraction (PXRD), as well as differential thermal analysis and thermogravimetric analysis and temperature programmed reduction (TPR), transmission electron microscopy (TEM), and N_2_ sorption. The catalytic properties were tested in a fixed bed reactor and benchmarked against an industrial‐type Ni/Al_2_O_3_ benchmark. The results show that a solid solution precatalyst with a very high surface area can be obtained, which is translated into a high dispersion of Ni in the reduced state. This leads to a high methane production rate at gas hourly space velocity applied in industry while being competitive or even outperforming the industrial reference catalyst.^[^ ^21^ ^]^ This study demonstrates the feasibility to derive a highly active methanation catalyst from a coprecipitated single‐source precursor and further improvements are to be explored in the future, e.g., by introduction of promoters. The facile nature of the synthesis and the abundance of the catalyst's raw materials holds promise for further development of highly active and stable Ni catalysts for this or for other reactions.

## 2 Results and Discussion

### 2.1 Catalyst Precursor Synthesis

The coprecipitation of a mixed Mg(NO_3_)_2_/Ni(NO_3_)_2_ solution with Na_2_CO_3_ solution at a constant pH and subsequent hydrothermal ageing yielded a nanocrystalline precursor with the sum formula (Ni_1–*x*_Mg_*x*_)_12_(CO_3_)_8_(OH)_6_O · *y* H_2_O (*x* = 0–0.5). The crystal structure of the pure basic nickel hydroxycarbonate (*x* = 0) was first published by Rincke et al.^[^ ^20^ ^]^ and found to be isotypic to the Mg‐substituted materials reported here.

**Figure 2:**
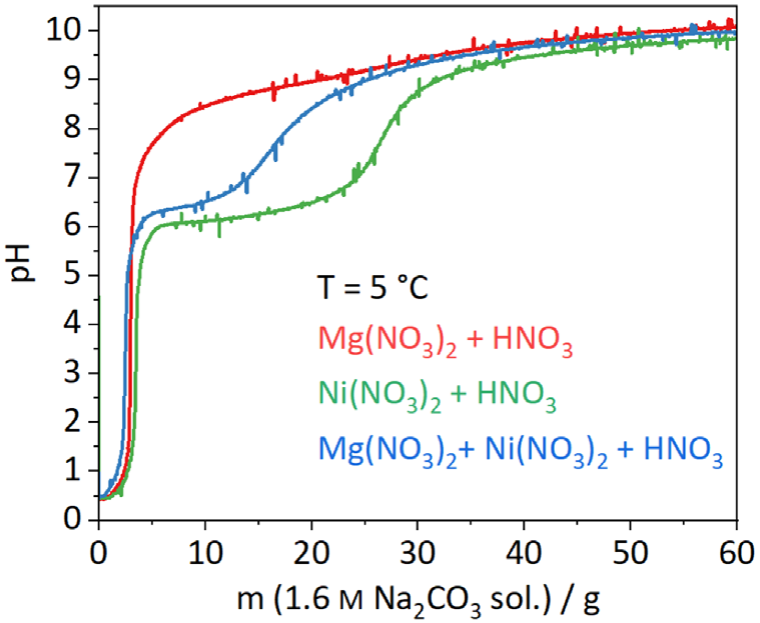
Titration curves of metal nitrate solutions against 1.6 M Na_2_CO_3_ solution at 5 °C.

To determine the optimal coprecipitation synthesis conditions, aqueous solutions of the different metal ions were titrated first individually and then simultaneously at different temperatures (5 °C, 25 °C, 60 °C) against the precipitating agent sodium carbonate solution (1.6 M). Therefore, 50 mL of a 0.5 M metal nitrate solution were placed in an automated reactor (for details, see Experimental section) and acidified with nitric acid to a starting pH of 0.5. The base was dosed with a rate of 1 g min^−1^ until the pH reached a plateau near the pH value of the sodium carbonate solution. Higher pH values could not be reached due to the only moderate basic strength of sodium carbonate. The titration curves recorded at 5 °C (chosen later as ideal precipitation temperature) are shown in **Figure** **2**; further data are shown in Figure S2, Supporting Information.

For titrating nickel nitrate against sodium carbonate solution, two plateaus (at pH 6 and 10) can be observed (see Figure S2, Supporting Information) with the slope of the step between the plateaus getting steeper as the temperature increases. The first refers to the precipitation of Ni, while the latter is the reaching of the final pH in an excess of base. The precipitated samples were X‐ray amorphous.

The titration of magnesium nitrate solution shows two plateaus at 60 °C (see Figure S2, Supporting Information) as well which are less pronounced at room temperature and morph into one at 5 °C. The first starts around pH 8.5 and refers to the precipitation of Mg. The subsequent titration where both Ni and Mg were present in the same solution appears to be a combination of both titration curves not indicating initial formation of a mixed precipitate but rather subsequent precipitation of first Ni and then Mg. These phenomena have been intensively studied in an analogous manner by Behrens et al. for copper and zinc.^[^ ^15^ ^]^ To achieve a rather simultaneous solidification of both components, a constant pH of 9, closely above the highest precipitation plateau, controlled by simultaneous dosing of both solutions and a temperature of 5 °C were chosen since these conditions are sufficient to coprecipitate both ions.

For the precursor synthesis at constant pH (see Figure S1, Supporting Information), different higher and lower pH values have also been investigated. These resulted in nonphase pure products after hydrothermal aging and/or a difficult and unsteady pH control due to an unbalanced simultaneous dosing of metal salt solution and base to reach a too high or low pH. The precipitation temperature of 5 °C was chosen because it inhibits Ostwald ripening and therefore generally results in a smaller particle/cs.^[^ ^13^, ^22^ ^]^

The solids directly obtained by coprecipitation at the chosen conditions are X‐ray amorphous in all cases. Therefore, a hydrothermal aging step is required, in line with the reported synthesis routes by Rincke et al.,^[^ ^20^ ^]^ but at different temperatures and duration ( see Experimental section). It was also observed by ICP‐OES measurements that the set Mg concentration did not correspond to the concentration in the precipitated solid, which had been separated from the mother liquor, when interrupting the synthesis before the hydrothermal aging step indicating that substantial amounts of magnesium are still present dissolved in the mother liquor. Therefore, after the coprecipitation, the entire slurry of the reaction mixture including the solid and the mother liquor was transferred into the autoclave. This slurry contains all of the dosed amounts of nickel nitrate, magnesium nitrate, and sodium carbonate solutions. The Mg concentration in the solid increased with hydrothermal aging time and asymptotically approached the target value, which was investigated by a series of termination experiments. It can therefore be concluded that during the hydrothermal ageing the magnesium ions are gradually incorporated into the solid from the mother liquor. However, small deficits between the nominal and the experimentally determined Mg compositions might occur for the Mg‐richer samples. In the following the nominal relative Ni and Mg content will be used to label the samples for simplicity. Nevertheless, the composition values used for the graphical representations refer to the measured elemental ratios. For example, a sample with a nominal ratio of metal cations of 70% Ni to 30% Mg is labeled NM7030, even if the measured value is 70.32% Ni or 29.05% Mg. The measured values for all precursor compositions can be found in Table S1, Supporting Information.

**Figure 3:**
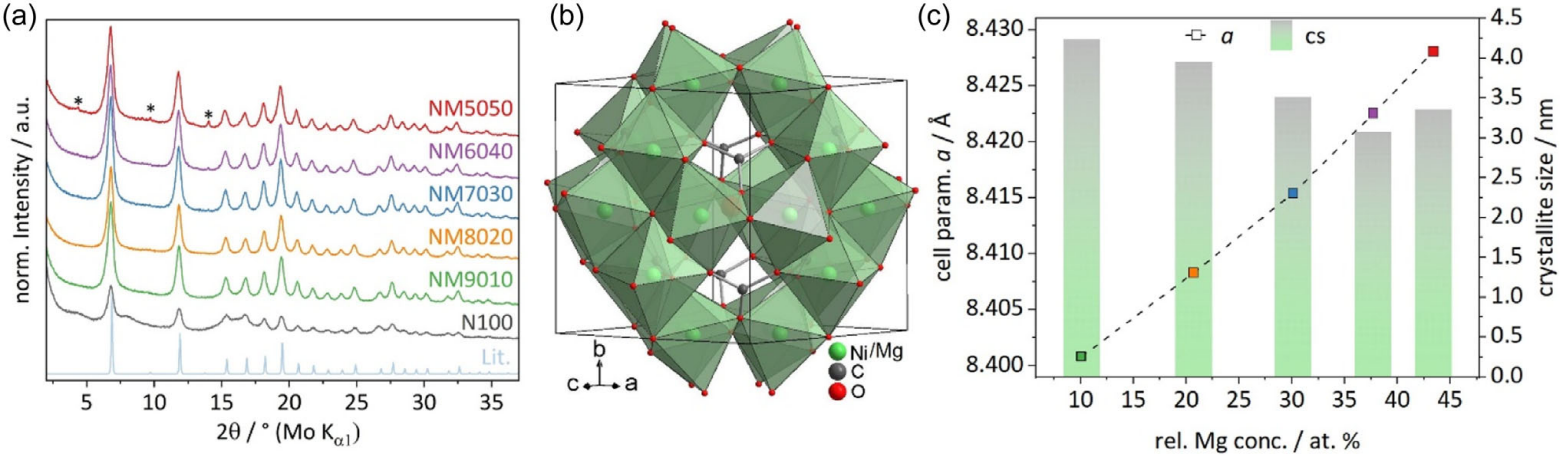
a) PXRD patterns of precursor samples from 0–50% nom. Mg content and literature reference,^[^ ^20^ ^]^ hydromagnesite side phase marked with stars, b) unit cell of crystal structure which is applicable for all (Ni_1–*x*_Mg_*x*_)_12_(CO_3_)_8_(OH)_6_O (*x* = 0–0.5), and c) calculated cell parameter (*a*) and average cs for samples of different rel. Mg concentrations.

During the hydrothermal ageing step, a bright green product crystallizes in the cubic space group $P\bar{4}3m$ (215). The crystal structure (see **Figure** **3**b) was solved and described in depth by Rincke et al.^[^ ^20^ ^]^ PXRD patterns for the samples in the compositional range of 0–90% Mg can be found in Figure S3, Supporting Information. A precipitation of a pure Mg solution, however, yielded in a mixture of hydromagnesite and brucite. Hydromagnesite occurs as a side phase with visible PXRD peaks in samples with Mg concentration of 90–50%, thus complicating the analysis of the substitution chemistry of the targeted phase in this range. Only with the help of Rietveld refinement, small side phase fractions of 3.12% and 14.6% could be determined also in the NM6040 and NM5050 samples. The presence of a significant proportion of crystalline MgO in all samples was excluded by Rietveld refinement.^[^ ^23^ ^]^ The difference plot of the refinement of the phase‐pure NM7030 precursor sample is shown exemplary in Figure S4, Supporting Information.

Since the ICP results in combination with no further shifts of peaks in the PXRD patterns (see Figure S5, Supporting Information) indicate that a substitution of Ni by Mg is only feasible up to 50%, it was decided to only proceed with the phase pure (NM9010, NM8020, NM7030) samples as well as the NM6040 and NM5050 samples with only low amounts of side phase in agreement with the goal of synthesizing highly loaded catalysts. For those samples, whose PXRD patterns are shown in Figure 3a, the refinements show a small cs in the range of 3–4 nm. There is a slight decrease in cs (Figure 3c) with increasing Mg content as well as an almost linear increase of the cell parameter *a* of the cubic lattice, which again confirms the substitution and thus the atomic distribution of both cations in the same sublattice. Despite the trend in cs, the incorporation of Mg generally seems to have a positive influence toward long range periodicity and crystallinity as the pure nickel compound (N100) exhibits poor crystallinity whereas products with already the lowest amount of added Mg show a larger domain size.

In a N_2_ physisorption experiment, the precursor materials exhibit a type IV(a) isotherm with an additional H2(b) hysteresis for NM7030 and Mg‐richer samples. For NM8020, the hysteresis of the isotherm represents an intermediate stage toward a H2(a) type, which is also observed for NM9010 and N100. The derived BET surface areas (analysis method of Brunauer, Emmett and Teller (BET)) of the precursors are large and correlate positively with the incorporated amount of Mg^2+^ increasing from 209 to 230 m^2^ g^−1^ (see **Table** **1**).

**Table 1:** Decomposition temperatures determined by the first derivative of the thermogravimetric curve as well as a comparative listing of BET surface areas for various material stages.

	N100	NM9010	NM8020	NM7030	NM6040	NM5050
Nom. Mg^2+^ content [%]	0	10	20	30	40	50
Decomposition temperature [°C]	289	334	343	356	362	364
Precursor BET S_A_ [m^2^ g^−1^]	289	209	217	222	225	230
Calcineda) BET S_A_ [m^2^ g^−1^]	31	72	136	184	152	166
Calcinedb) BET S_A_ [m^2^ g^−1^]	–	128	–	229	–	299

a) Samples calcined for 12 h at 400 °C.

b) Samples calcined for 3 h at 400 °C.

### 2.2 Calcination and Solid‐Solution Precatalyst

**Figure 4:**
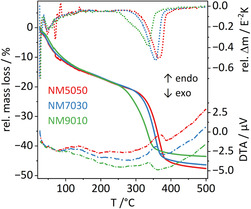
TG (full lines), DTG (dotted lines), and DTA (dash dotted lines) profiles for temperature resolved decomposition in air of for samples with varying Mg^2+^ concentrations.

To maintain the small cs, the decomposition process of the precursor needs to be optimized regarding calcination temperature and time and was studied in detail. As it can be seen in DTA/TG measurements (**Figure** **4**), the decomposition temperature rises with increasing Mg content in the samples. Due to the lower atomic mass of Mg and different amounts of crystal water, the relative total mass loss varies, but the shape of TG and DTA curve is very alike for samples with different Mg contents. The gradual mass loss near 100 °C can be assigned to the evaporation of adsorbed and crystal water while the crystal structure stays intact (see temperature‐resolved PXRD, vide infra). The following mass loss indicates the decomposition of the structure accompanied by release of CO_2_ and H_2_O to form the remaining oxide, as also previously reported for the pure nickel compound^[^ ^20^ ^]^ based on a combined TG‐IR experiment. The decomposition temperature for our mixed samples with various Mg^2+^ contents is listed in Table 1 as the peak temperature of the DTG curves and found to be lower than reported for the pure nickel compound, but in good agreement with the in situ PXRD thermal decomposition experiment shown in Figure 6b. The postulated equation for the thermal decomposition reaction is given in Equation (2).

(2) $${\left({\text{Ni}}_{1–x}{\text{Mg}}_{x}\right)}_{12}{\left({\text{CO}}_{3}\right)}_{8}{\left(\text{OH}\right)}_{6}\text{O }\to \text{ 12 }\left({\text{Ni}}_{1–x}{\text{Mg}}_{x}\right){\text{O + 8 CO}}_{2}{\text{+ 3 H}}_{2}O$$

In an in situ PXRD thermal decomposition experiment, NM7030 (Figure S11, Supporting Information) and NM5050 (Figure 6b) show the same interesting behavior as the pure N100 compound.^[^ ^20^ ^]^ The reflections of (222), (004), (044) of the precursor material, which are transformed in (–111/111, 020, –202/022), in the calcined product stay in the same position (apart from thermal expansion of the lattice). With the here investigated substitution, it could be confirmed that Ni and Mg can be located at the same site in the precursor, as was previously proposed by Rincke et al., supporting their proposed mechanism of thermal decomposition and oxide formation.^[^ ^20^ ^]^

**Figure 5:**
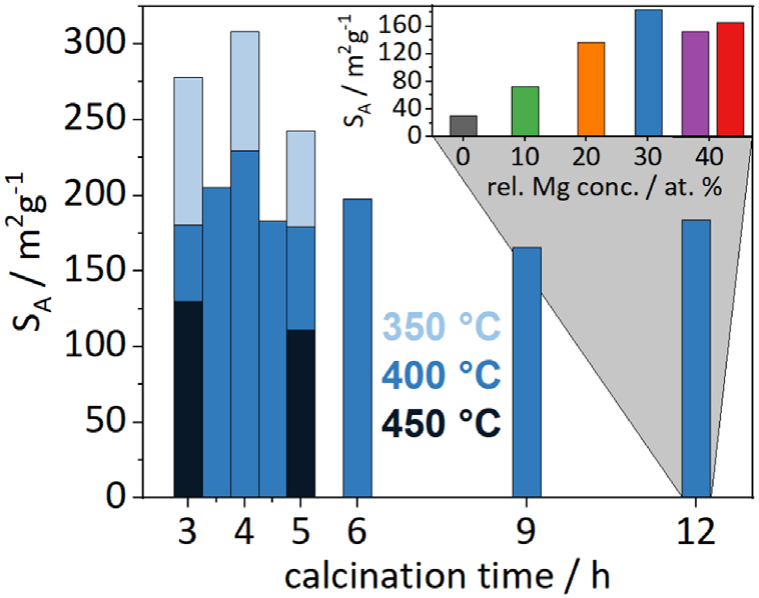
Graphical representation of the BET surface area in dependence of calcination time and temperature of for samples with 30 at.% Mg (nominal, NM7030) (top right corner) BET surface area in dependence of Mg concentration at a calcination time of 12 h at 400 °C.

**Figure 6:**
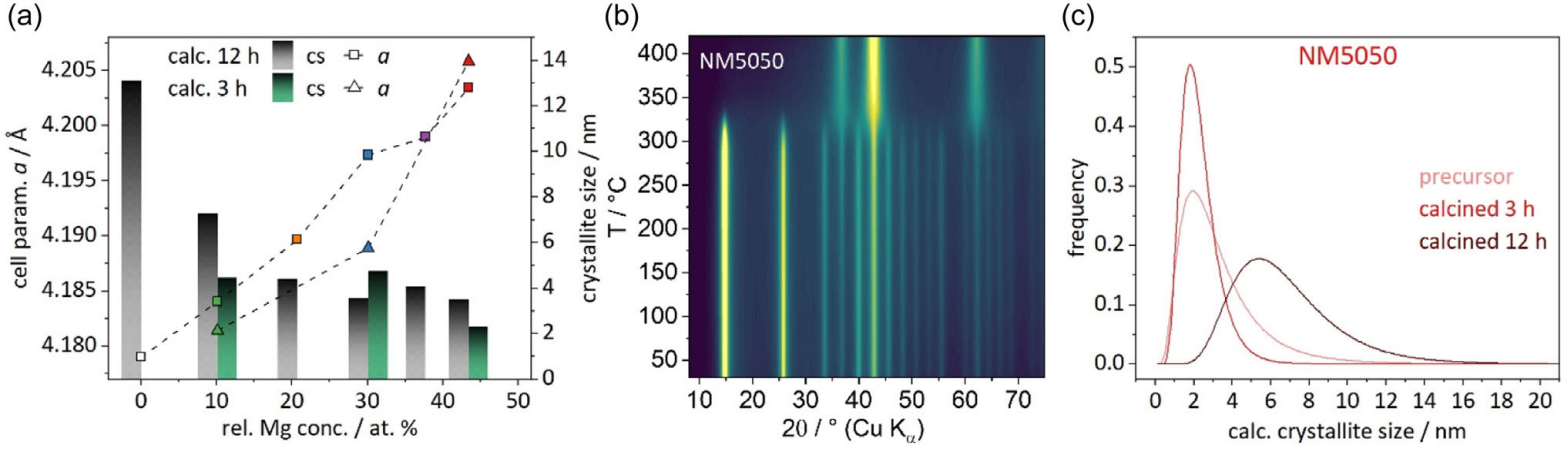
a) Calculated cell parameter *a* and average cs for samples of different Mg concentrations calcined for 3 or 12 h at 400 °C. b) Temperature‐resolved PXRD experiment on NM5050 in static air. c) Domain sizes derived from WPPM calculations on the precursor and samples of NM5050 calcined at 400 °C.

The calcination temperature affects the diffusion coefficient and impacts sintering and thus the specific surface area. Samples were taken from one precursor batch with a nominal magnesium content of 30% (NM7030) and heated to 350, 400, or 450 °C (as derived from DTG) within 1 h and calcined for 3–12 h at the target temperature. Even after calcination over 12 h at 400 °C, still a surface area of 184 m^2^ g^−1^ was observed. Calcining for a shorter time (3 h) resulted in broadened peaks in the XRD patterns which indicate a lower cs, which decreases from 6 to 2 nm (see Figure 6c). By lowering the temperature down to 350 °C, broad and nonspecific signals can be observed in the PXRD patterns at diffraction angles where the precursor exhibits peaks, which hints toward an incomplete decomposition. The samples were tested for BET surface area (see **Figure** **5**) where the highest value of 308 m^2^ g was observed for a calcination program of 350 °C for 4 h. The adsorption isotherms can be found in Figure S9 and S10, Supporting Information.

A significant amount of residual carbon (>1.5 wt%) was determined by elemental analysis, pointing potentially to an incomplete decomposition of the carbonates, which is supported by IR spectroscopy (see Figure S8, Supporting Information). Finally, a calcination temperature of 400 °C was chosen since there was less residual carbon (<1%). For higher calcination temperatures, the carbon value content is even lower but the surface area decreases accordingly. This is rather caused by an effect of thermal sintering than by a stabilizing influence of carbon since the surface area does not correlate in a clear trend with the carbon content. Therefore, and for the findings from the XRD studies mentioned above, a standard calcination temperature of 400 °C for 3 h was chosen.

Since the isotherms and hysteresis do not change their shape when comparing the precursor and calcined samples (Figure S8, Supporting Information), it can be assumed that the general texture stays intact over the calcination process and that the alterations of the BET surface area are due to the competing effects of particle shrinking and density increase as well as sintering. The experimental data (Figure S8, Supporting Information) for the adsorption follow the characteristic type IV(a) isotherm shape. The hysteresis feature changes with varying Mg content showing a type H2(b) hysteresis for a nominal Mg content of more than 30%, while it is of type H2(a) for the samples with nominal Mg content of 10–0%. At a nominal Mg content of 20%, the isotherm is intermediate between these two types. This indicates complex network structure pores of 4 nm and larger partly blocked pores.^[^ ^24^ ^]^

As expected from the similar ionic radii^[^ ^25^ ^]^ and in line with previous literature reports,^[^ ^26^, ^27^ ^]^ a mixed metal oxide Ni_1–*x*_Mg_*x*_O is formed, which is further supported by the elemental mapping carried out in the TEM (vide infra). The PXRD patterns are shown in **Figure** **6**b, corroborating the formation of phase pure mixed oxides. The changes in the lattice parameters (derived by Rietveld refinements, see Figure 6a) and in the crystallite sizes are indicative for the formation of mixed crystals: With increasing Mg content the lattice parameter of the cubic halite type structure increases, as expected from the lattice parameters of the pure oxides (NiO^[^ ^28^ ^]^ *a* = 4.17610(4) Å, MgO^[^ ^29^ ^]^ *a* = 4.22 Å). Furthermore, the apparent cs decreases with increasing Mg content. This may stem from a hampered crystallite growth due to the substitution or an additional strain‐like component due to the substitution, which was not accounted for here. A more elaborate way to analyze the cs is by means of whole powder pattern modeling (WPPM), which allows to extract information on the cs distribution shown in Figure 6c. A comparison of the probability density functions (here using NM5050 as example) shows that the maximum of the size distribution is constant after a short calcination period and shifts to larger sizes after a long calcination period. The width of the distribution initially becomes narrower and then significantly wider. From this, it can be concluded that the domains of the solid solution grow by sintering as the temperature is maintained for longer times. For NM7030 and NM9010, the size distributions can be found in Figure S6, Supporting Information.

### 2.3 Active Catalyst and Microstructure

**Figure 7:**
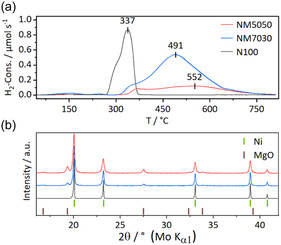
a) H_2_‐TPR profile of oxide precatalysts with different Ni:Mg ratios with marked temperature maxima of H_2_‐consumption. b) XRD patterns of these samples after reduction with references of Ni and MgO.^[^ ^29^, ^47^ ^]^

To bring the catalyst to its active state, it has been reduced with diluted hydrogen at elevated temperatures to trigger a segregation of Ni nanoparticles from the mixed nickel and magnesium oxide. The optimal reduction temperature was determined by a TPR up to 800 °C (in 5% H_2_ in Ar). For each sample, the TPR curve exhibits a main peak of hydrogen consumption shouldered on the left besides a weak signal at low temperatures (**Figure** **7**a). The latter can be interpreted as reduction of low amounts of nonstochiometric Ni(III) since the material undergoes a color change at this temperature (see SI). Further, the peak shoulder refers to the reduction of Ni(II) in the first few accessible surface layers.^[^ ^30^ ^]^ Consequently, the main peak results from bulk reduction. With a rising Mg content in the sample, a strong broadening of the TPR peaks and a shift to higher temperatures compared with the pure Ni sample can be observed. In general, the peak broadening may be related to some extent to the crystallites becoming larger, as shown before with Rietveld refinement (see Figure 6a). With larger particles, the reducibility of the internal atoms is hindered by longer diffusion paths. Moreover, there are apparently strong interactions between nickel, oxygen, and magnesium in the solid solution oxide, so that the former becomes less reducible if surrounded by Mg^2+^ rather than other Ni^2+^ in the second coordination shell, leading to a shift of the reduction maximum to higher temperatures.^[^ ^26^, ^27^ ^]^ The reaction equation for the complete Ni reduction in hydrogen is given in Equation (3):

(3) $$\left({\text{Ni}}_{1–x}{\text{Mg}}_{x}\right)\text{O + 1}–x{\text{ H}}_{2}\to \;x\text{MgO + 1}–x\text{Ni+1}–xH_{2}O$$

Subsequent PXRD analysis of the samples after the TPR experiment was performed in sealed capillaries under the exclusion of air and confirm a complete reduction of Ni. In samples with the exception of N100, the unreducible MgO phase is still present in proportion. No peaks matching crystalline metallic Mg or nickel oxide could be observed. Also, the hydrogen consumption matches a degree of nearly complete reduction of Ni after a TPR up to 800 °C. However, an isothermal reduction at 500 °C of NM7030 for the catalytic experiments shows in a Rietveld refinement a proportion of nickel which remains unreduced in the solid solution (see Figure S12, Supporting Information).

**Figure 8:**
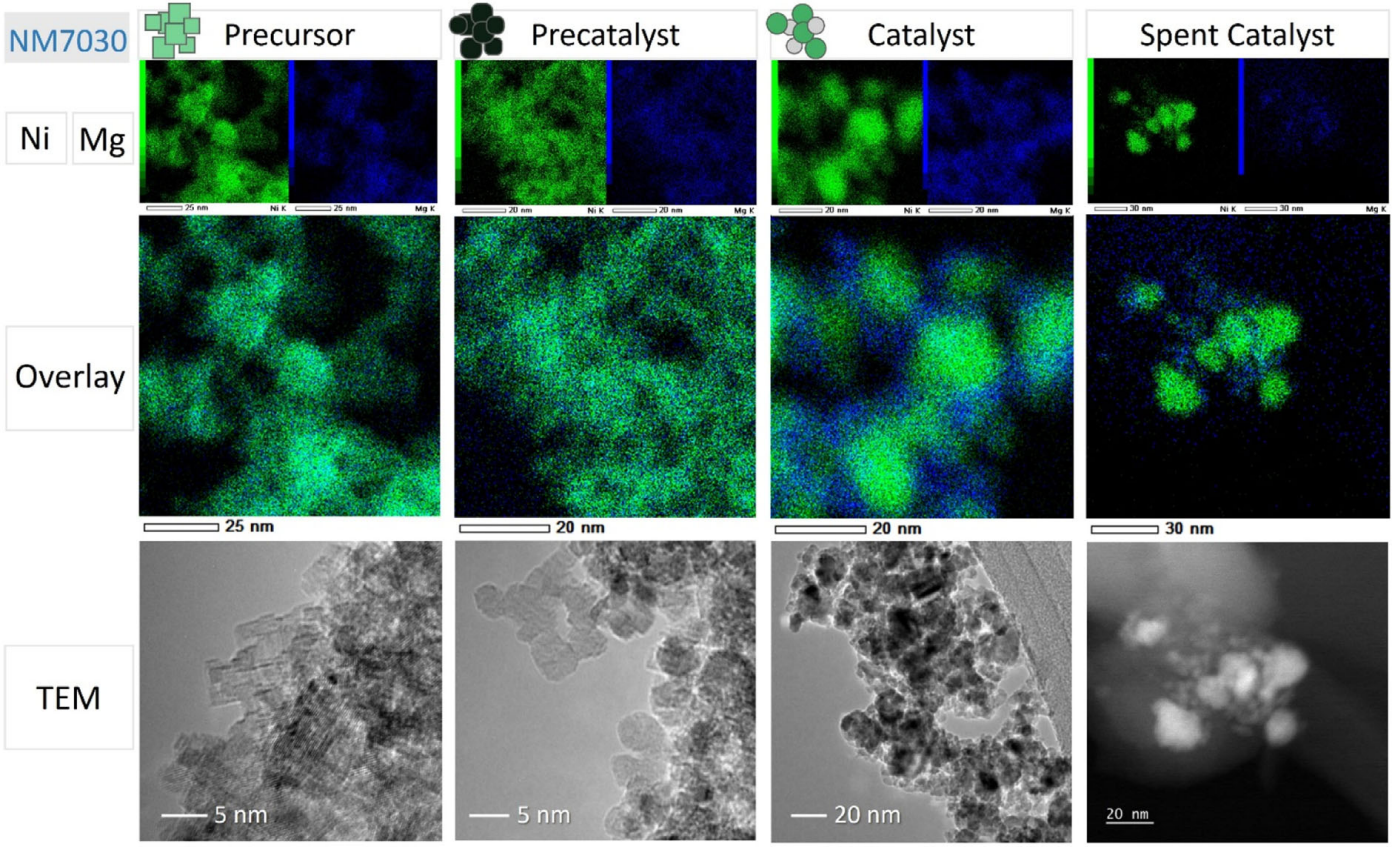
TEM imaging and EDX mappings of different stages of the catalyst: precursor (Ni_0.7_Mg_0.3_)_12_(CO_3_)_8_(OH)_6_O, precatalyst (Ni_0.7_Mg_0.3_)O, catalyst (passivated) Ni/MgO, spent catalyst (passivated). Precursor, precatalyst, and catalyst are pictured in bright field mode; the spent catalyst in annular dark field mode (ADF‐STEM). Green, nickel; blue, magnesium.

Particle and crystallite morphology and size were determined for different stages of the catalyst synthesis by transmission electron microscopic (TEM) analysis for different states of the NM7030 sample as an example. The precursor (**Figure** **8**) contains crystallites shaped as cubes or in a cuboid fashion not exceeding 7 nm in edge length in good agreement with the cs calculated from XRD by the WPPM approach. Elemental mapping revealed a homogeneous distribution of the elements on an atomic level as expected for a mixed salt.

After calcination, the particles appear to be 5 nm or larger in diameter. The calcination leads to particles with an overall less defined shape. Some show sharp edges, while others are of rounded shape. Nickel and magnesium still are homogeneously distributed on an atomic level, which was proven by elemental mapping and support the interpretation of the corresponding PXRD patterns regarding the formation of a solid solution.

The TEM images shown for the activated catalyst stem from a reduced and afterward passivated sample since no inert transfer into the microscope was available. Presumably, only a thin passivating oxide layer of NiO formed on the metallic Ni surface. This is supported by PXRD analysis which showed little to no evidence of NiO but intense peaks of metallic Ni. The Ni particles reach a size of up to 20 nm, while MgO forms much smaller crystallites surrounding and thus stabilizing Ni as can be seen in the elemental mapping.

The spent resp. postmortem catalyst shows a highly similar morphology to the freshly reduced and passivated catalyst. The domain size of nickel crystallites has increased but does not exceed 30 nm in diameter. Agglomerates of MgO are still separating the Ni particles. From the series of TEM images, it can be concluded that the microstructure of the active catalyst can be described as porous aggregates of Ni nanoparticles, which are spatially separated by MgO particles preventing them from sintering. The porosity originates from alternating arrangement of the two components rather than from their intrinsic porosity. This microstructure, which is similar to the industrial Cu/ZnO catalyst, evolved during activation from the solid solution nanoparticles and remained rather stable during the reaction as only minor particle growth was observed.

### 2.4 Catalytic Performance

The catalysts NM5050, NM7030, and NM9010 were tested in the Sabatier reaction (Equation (1)) from 240 to 280 °C in steps of 10 °C at atmospheric pressure and at elevated pressures (5, 10, 15 barg) at 260 °C. Selectivity to methane and carbon monoxide formed in the competing rWGS (reverse water gas shift) reaction (Equation 4) was monitored.
(4)

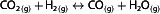




As a benchmark material, an industrial type catalyst (Ni on aluminum oxide—referred to below as “SPP2080‐IMRC”) with 8.3% Ni was chosen. The name “SPP2080‐IMRC” stands for “Industrial Methanation Reference Catalyst” of the SPP2080 priority program^[^ ^31^ ^]^ of the German Research Foundation (DFG). This SPP2080‐IMRC catalyst was previously studied in detail by Weber et al.^[^ ^21^, ^32^, ^33^ ^]^ In contrast to the catalyst presented herein, the benchmark material uses aluminum oxide instead of magnesium oxide as a support and has a relatively low nickel loading of ≈8.3 wt%.^[^ ^21^, ^31^ ^]^ Its microstructure can be assumed to resemble more to a typical impregnated catalyst with a preformed intrinsically porous support and Ni nanoparticles decorated on outer surface and the inner pore walls. The same reduction conditions (500 °C (773 K), 0 barg, 5% H_2_ in N_2_, 25 sccm, holding time 3 h, ramp 5.5 K min^−1^, mass ≈10 mg (for details see Table S4, Supporting Information)) were chosen for all catalysts based on the TPR of the precatalysts (vide supra) and on literature values for the SPP2080‐IMRC.^[^ ^21^ ^]^ For loading the reactors, the benchmark catalyst which was in the form of spheres (Ø 1.5 mm) needed the be crushed and then sieved to a particle size fraction of 100–200 μm just like the catalyst obtained via the precursor route accordingly. The reduction properties of spheres in contrast to crushed powder of this catalyst have been investigated by Weber et al.^[^ ^12^ ^]^ as well, and it was found that the powder shows two main reduction peak maxima at 269 °C (542 K) and 527 °C (800 K).^[^ ^12^ ^]^ Additional information on the state of the NM7030 catalyst after isothermal reduction is exemplary given in Figure S12, Supporting Information. The catalytic testing followed immediately after the reduction (for device details, see Characterization). During the reaction series, a gas flow of 4.75 sccm CO_2_, 19 sccm H_2_, and 1.25 sccm He as internal standard was applied to each reactor. The product gas stream was further diluted with 20 sccm N_2_ before GC sampling.

**Figure 9:**
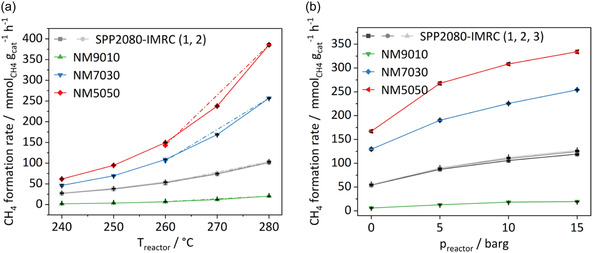
a) Methane formation rate for various catalysts at constant pressure of 0 barg. The catalysts were tested stepwise and after reaching the maximum step again at the midpoint of the scale to test stability. b) Methane formation rate at a constant temperature of 260 °C. The reference catalyst was measured in multiple runs (marked 1,2,(3) each), the results of which coincide so that they lie on top of each other.

As shown in **Figure** **9**, the methane formation rate based on catalyst mass increases with pressure and exponentially with temperature for all catalysts. The industrial benchmark catalyst was measured multiple times (labeled SPP2080‐IMRC 1, 2, 3) and the results confirm a low error of the catalytic measurements. After reaching the maximum of 280 °C the temperature was lowered again to 260 °C to test stability, which showed no or only minor deactivation in methane production rate due to sintering or coking for all tested catalysts (see Figure S13, Supporting Information). A higher MgO fraction in the NM catalysts leads to higher rates. This can be explained with the dispersing effect that MgO exerts on the Ni component. At low Mg content of 10%, the Ni nanoparticles formed from the solid solution precatalysts can grow to a relatively large size, therefore exposing a lower metallic surface area and a lower amount of potentially active interface or perimeter sites^[^ ^3^, ^34^ ^]^ of reduced metal and oxide support in NM9010. As seen in the TEM study on NM7030, a higher Mg content leads to an effective interdispersion of both phases, which can be assumed to be maximized around 50% Mg in NM5050, which is by far the most active catalyst in this study, likely due to a higher metal surface area and/or more points of contact between Ni and MgO. At a temperature of 280 °C, the NM5050 outperforms the industrial catalyst by a factor of ≈4. The carbon balance approaches 100% in almost all experiments (see Table S2 and S3, Supporting Information). Under pressure the catalysts exhibit an expected behavior of increasing methane formation rate with rising pressure. Due to the dilution of the catalyst bed with silicon carbide and the associated distribution over the isothermal zone, the space velocity (≈150 Lg^−1^ h^−1^) is calculated in relation to the total mass and not the volume of the catalyst.

**Figure 10:**
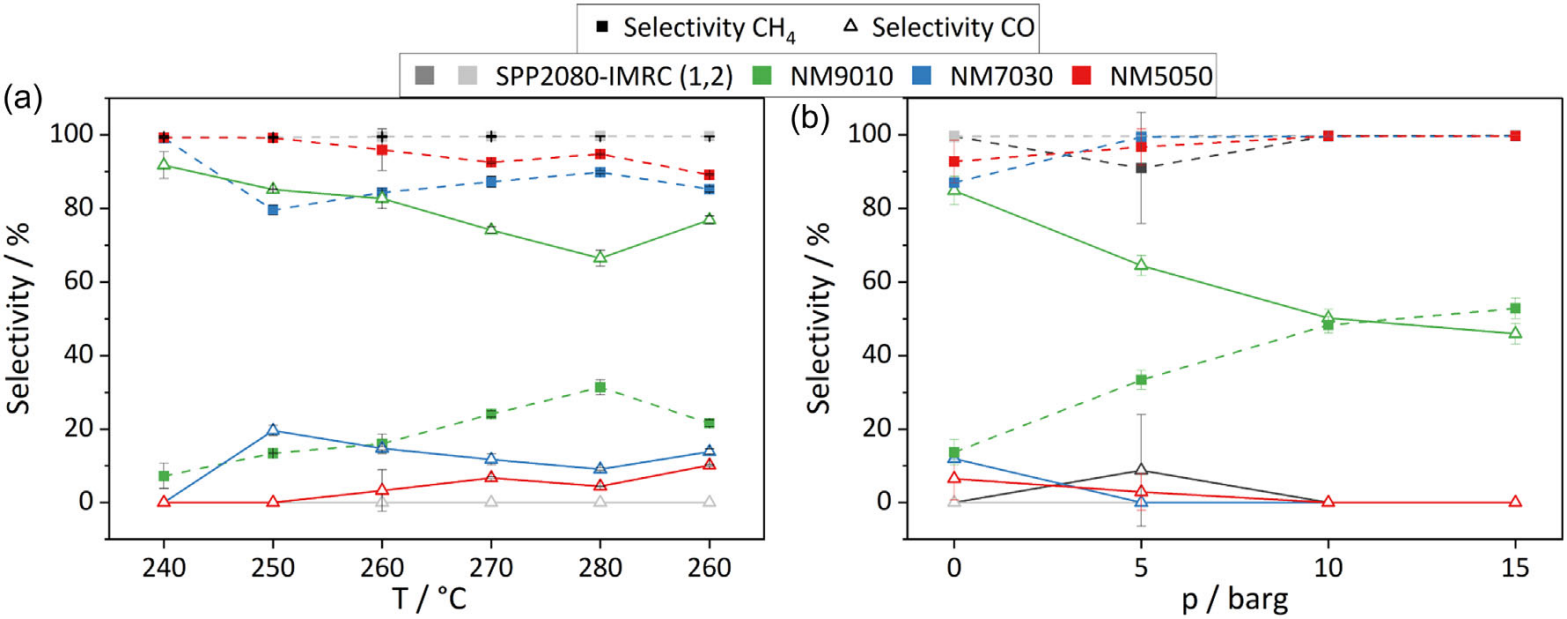
a) Selectivity toward methane and carbon monoxide for various catalysts. The catalysts were tested stepwise from 240 °C up to 280 °C and afterward again at 260 °C to test stability at 0 barg. b) Selectivity toward methane and carbon monoxide for pressures from 0 to 15 barg at 260 °C. The reference catalyst was measured in two runs (marked 1,2).

Regarding selectivity, over the industrial catalyst, NM7030 and NM5050, the Sabatier reaction dominates at all pressures and temperatures with a selectivity of 80–100% toward methane (**Figure** **10**). No strong trend of selectivity with reaction temperature is observed for these three catalysts. Only for the NM9010 catalyst the rWGS is favored at all temperatures and the selectivity to CO is increasing with temperature as expected from thermodynamics. Also, the pressure shows a clear effect on selectivity for this catalyst. Higher pressure favors methanation over rWGS, which becomes the dominant reaction over NM9010 at pressures over 10 barg. All other catalysts show only minor selectivity variations with pressure and maintain their high selectivity to methane. It can be speculated that the higher selectivity of NM5050 and NM7030 compared to NM9010 is due to another positive effect of the MgO support beyond mere stabilization and may originate from the contact area of the two components. In NM9010, the nickel particles are largest and the catalyst is expected to expose a large fraction on nickel surface area that is not affected by the support, while in NM7030 and NM5050 the nickel particles are smaller and better interdispersed with the support phase. Thus, the exposed nickel surface area can be affected stronger by interaction with MgO. Also, the favorable aspects of ex‐solid solution catalysts mentioned in the introduction can be assumed to be more prevailing in the higher Mg‐loaded catalysts (see Figure S14, Supporting Information).

Mechanistically, CO_2_ methanation on Ni/MgO was proposed to proceed via the formate route (HCOO*) based on DFT calculations.^[^ ^34^ ^]^ CO_2_ adsorbs preferentially on the basic (Ni)MgO surface. Hydrogen adsorbs and dissociates on the metallic Ni particles. The hydrogen atoms can spillover to the support creating a synergistic effect promoting CO_2_ hydrogenation.^[^ ^34^ ^]^ The favorable interaction of the two components and the high number of active sites are thus likely reasons for the good performance in line with the observed trends.

Further products (apart from methane (CH_4_) and carbon monoxide (CO)) are trace amounts of ethane (C_2_H_6_) (<2.0%), methanol (CH_3_OH) (<0.5%) (see Figure S15 and 16, Supporting Information), propane (C_3_H_8_) (<0.05%), and ethanol (CH_3_CH_2_OH) (<0.05%) (not shown). These by‐products occur significantly more frequently with the NM catalyst to an increasing extent with decreasing magnesium content. C_2+_ products such as ethane have been recently described by Monai et al. and may originate from strong metal–support interactions on Ni/TiO_2_.^[^ ^35^ ^]^ MgO is a rather irreducible support and here, these C–C coupling products might rather be due to a low activity of MgO for oxidative coupling of methane to ethane, while Al_2_O_3_ in the industrial benchmark exhibits that behavior to a much lesser extent. In the absence of oxygen, one possible reaction mechanism is the creation of CH_3_* radicals by MgO polarizing methane over Mg–C and H–O interaction.^[^ ^3^ ^]^ This scenario is in line with the smallest and thus possibly most reactive MgO particles in NM9010 compared to the Mg‐richer NM catalysts.^[^ ^36^ ^]^ The second most abundant by‐product is methanol, which supports the formate route as HCOO* species are known precursors for methanol.

## 3 Conclusion

In this work, we presented the synthesis of a novel highly active Ni/MgO methanation catalyst capable of stabilizing high metal loadings. Its microstructure is intermediate between a skeletal Ni and a classical impregnated Ni catalyst and resembles that of industrial Cu/ZnO catalysts for methanol synthesis. Indeed, the well‐established synthetic concept from a crystalline coprecipitated precursor^[^ ^13^ ^]^ has been successfully transferred to the Ni–Mg system maintaining its strength of facile and in principle scalable preparation of a highly interdispersed state of the active metal and supporting oxide. Unlike the previously reported Ni–LDH precursors,^[^ ^37^ ^]^ the new hydroxy carbonate precursor compound (Ni_1–*x*_Mg_*x*_)_12_(CO_3_)_8_(OH)_6_O · *y* H_2_O can be obtained by hydrothermal ageing and gives rise to phase‐pure Ni_1–*x*_Mg_*x*_O solid solutions upon calcination. Control over composition is obtained by the partial substitution of Ni^2+^ by Mg^2+^ in the precursor, which was established for *x* up to 0.5. A substitution degree higher than 50% could not be achieved, which is reflected in no further expansion of the lattice parameter, while at the same time hydromagnesite occured as a side phase. Already the precursor is a nanostructured powder with crystallite sizes of less than 10 nm and exceptionally high BET surface areas in excess of 200 m^2^ g^−1^. The latter can be preserved to some extent after mild calcination and formation of mixed Ni_1–*x*_Mg_*x*_O; here the dilution with Mg^2+^ clearly retains higher surfaces areas of up to 184 m^2^ g^−1^ whereas the pure Ni compounds exhibit only 31 m^2^ g^−1^ after treatment at 400 °C and 12 h. Shorter calcination times preserve even higher surface areas, partly in excess of 200 m^2^ g^−1^.

The reduction temperature is again highly composition‐dependent, which is reasonable as the phase separation from the mixed oxide is less facile compared to pure NiO. In the methanation reaction, the catalysts with 70% and 50% Ni loading outperform an industrial benchmark catalyst, while 90% Ni loading leads to inferior performance. Thus, higher rates come with better interdispersion of the active metal and the support expected at equimolar ratio in the single‐source precursor and precatalyst.

This already competitive performance may be further enhanced by fine‐tuning of the Ni:Mg ratio and more rigorous optimization of the synthesis parameters. Promotion by other metals (e.g., Fe) may foster an even higher catalytic performance.^[^ ^38^, ^39^ ^]^ Furthermore, the reduction procedure offers potential for optimization, as the best reduction temperature to balance maximum reduction and sintering is composition‐dependent, which was not exploited in this study for the sake of comparability in our catalytic testing.

To conclude, Ni/MgO catalysts derived from this particular single‐source precursor are highly active, selective, and stable and the synthesis route holds great promise for further optimization for application in methanation as well as for fundamental studies of the role of Ni–Mg interactions in this and other reactions.

## 4 Experimental Section

4.1

4.1.1

##### Materials

The following materials were used for coprecipitation without further purification: nickel nitrate hexahydrate (abcr, 99.9%), magnesium nitrate hexahydrate (Merck, 99%), water‐free sodium carbonate (Grüssing, 99.5%), and nitric acid 65% (Honeywell, p.a.).

##### Synthesis

The precursor synthesis was carried out in an EasyMax 102 (up to 100 mL) or an OptiMax (up to 1 L) (both by Mettler Toledo), which were automated laboratory reactor systems. They were equipped with a glass reactor embedded in a solid‐state thermostat. The parameters such as temperature, stirring speed, and pH were adjusted and monitored by a computer controlled regulatory cycle. The 1 M acidic metal salt solution (25 g for EasyMax batch, 100 g for OptiMax batch) with the desired Ni:Mg ratio was gravimetrically dosed with a rate of 1 g min^−1^ by membrane pumps into the prefilled reactor (50 mL for EasyMax batch, 200 mL for OptiMax batch of demin. water). The precipitating agent (1.6 M Na_2_CO_3_ solution) was dosed simultaneously by a membrane pump as well so that the pH was kept constant at a value of 9 at a temperature of 5 °C. The volume strokes of the pumps were set to a ratio of 1:2 (acid:base). The stirring speed was kept constant at 300 rpm.

For hydrothermal ageing, the whole reaction mixture was transferred to Teflon lined steel autoclaves immediately (4 × 37 mL for EasyMax batches, 1 × 500 mL for OptiMax batches, filled ≈2/3 each) and then sealed. After ageing at 100 °C in a preheated furnace for 20 h (natural cooling), all samples were washed thoroughly with deionized water via repeated centrifugation and redispersing until the conductivity of the centrifugate was below 100 μS cm^−1^ and were afterward dried at 60 °C in air overnight directly in the centrifuge tubes. A bright green powder was obtained after being ground in an agate mortar with minimal force.

The calcinations were carried out in a muffle furnace in glazed ceramic crucibles with 1–2 g per crucible with a heating ramp of 6.7 °C min and a holding period of 3 h at the target temperature of 400 °C if not specified otherwise. The calcined material was then cooled at a natural cooling rate inside the muffle furnace.

##### Characterization: PXRD

If not stated otherwise, the samples were prepared as flat layers and characterized by PXRD. The patterns were recorded with a laboratory powder diffractometer Stadi P (STOE); Cu K_α1_ or Mo K_α1_ radiation, Johann‐type Ge (111) monochromator, Mythen 1 K detector (Dectris) in transmission geometry. For the reduced samples and Rietveld refinements, the measurements were executed on the same device in Debeye–Scherrer geometry with the sample loaded in a rotating sealed glass capillary.

The temperature‐resolved PXRD measurements were carried out on a Panalytical Empyrean diffractometer equipped with Ni‐filtered Cu K_α_ radiation and a PIXcel 1D detector in reflection geometry using an Anton Paar XRK 900 cell. The sample was held in static air; measurements were carried out during ≈20 min per temperature step.

Structure refinements were carried out using TOPAS Academic version 6.0. Instrumental line broadening was described using the fundamental parameter approach^[^ ^40^, ^41^ ^]^ as implemented in TOPAS and cross‐checked against a measurement of LaB_6_ (NIST SRM660c). Volume weighted domain sizes were calculated by the included routine as well.^[^ ^42^ ^]^ WPPM was used for crystallites with spherical form to determine the probability density function of crystallite sizes.^[^ ^43^ ^]^

##### Characterization: Elemental Analysis

For analysis of the elements C, H, N, and S, a Vario MICRO cube (Elementar) elemental analyzer was used. The content and ratio of alkali, alkaline earth, and transition metals were determined by an ICP‐OES Avio 200 (PerkinElmer).

##### Characterization: Thermal Analysis

Thermogravimetric measurements DTA/TG were carried out on a STA PT 1600 and STA 1000 (Linseis Messgeräte GmbH) equipped with a Pt/Rh‐thermo couple in flow of 100 sccm synthetic air.

##### Characterization: Sorption Analysis

The specific surface areas (by BET) of the powders were derived from N_2_ adsorption isothermal measurements carried out at a temperature of −196 °C on Belsorp MiniX or Belsorp Max instrument. Some data were obtained in a Nova 3200e sorption station from Quantachrome. Prior to data acquisition the samples were pretreated for 3 h at 180 °C at reduced pressures of 10–50 mbar (Quantachrome) or 0.1–1 mbar (Belsorp) to ensure desorption of water, CO_2_, N_2_, or other gases from the surface.

##### Characterization: Electron Microscopy

For the TEM measurements, the sample powder was dispersed in butanol and then dropped onto a copper TEM grid with a lacey carbon support (Plano GmbH). TEM images and electron diffraction pattern were recorded on a FEI Tecnai F30 G^2^ STwin (300 kV, FEG). ADF‐STEM micrographs and EDX elemental mapping were conducted on a probe Cs‐corrected JEM‐ARM200F NEOARM from JEOL (200 kV) equipped with two front and entry side positioned silicon drift EDX (100 mm^2^ area each) detectors.

##### Characterization: Catalytic Testing

The catalytic testing at pressures from 0 to 15 barg and elevated temperatures from 240 to 280 °C were performed in an Avantium Flowrence XD equipped with four parallel reactors in a fixed bed configuration and a downstream gas chromatograph analyzer. The reaction gas mixture was analyzed with an Agilent 8890 GC system with two sample loops. The instrument was equipped with two flame ionization detectors and two thermal conductivity detectors.

The precatalysts (sieve fraction 100–200 μm, ≈10 mg, see Table S4, Supporting Information) were diluted with SiC (≈50 mg) and loaded in a stainless steel reactor tube (inner diameter = 2 mm) and then pretreated in the device at 120 °C in a pure nitrogen flow to remove adhering gas molecules. The samples were reduced prior to the catalytic testing to form the active catalyst under the following conditions: ramp 5.5 K min^−1^ to 500 °C, holding time 3 h, 0 barg, 1.25 sccm H_2_, 23.75 sccm N_2_. Successive, the following reaction conditions were applied: ramp 5 K min^−1^, 4.75 sccm CO_2_, 19 sccm H_2_,1.25 sccm He, 240–280 °C (513–553 K), and 0 bar OR 260 °C (533 K) and 0–15 barg. The product gas stream was further diluted with 20 sccm N_2_ before GC sampling.^[^ ^44^, ^45^, ^46^ ^]^

## Conflict of Interest

The authors declare no conflict of interest.

## Supporting information

Supplementary Material

## Data Availability

The data that support the findings of this study are available from the corresponding author upon reasonable request.

## References

[1] C. Vogt , M. Monai , G. J. Kramer , B. M. Weckhuysen , Nat. Catal. 2019, 2, 188.

[2] M. S. Wainwright , Handbook of Heterogeneous Catalysis, Vol. 3, (Eds: G. Ertl , H. Knözinger , J. Weitkamp ), Wiley‐VCH, Weinheim 1997, pp. 66–67.

[3] R. Schlögl , Angew. Chem. Int. Ed. 2015, 54, 3465. 10.1002/anie.201410738 25693734

[4] Y. H. Hu , E. Ruckenstein , Catal. Lett. 1997, 43, 71.

[5] S. Rönsch , J. Schneider , S. Matthischke , M. Schlüter , M. Götz , J. Lefebvre , P. Prabhakaran , S. Bajohr , Fuel 2016, 11, 276.

[6] M. Younas , L. Loong Kong , M. J. K. Bashir , H. Nadeem , A. Shehzad , S. Sethupathi , Energy Fuels 2016, 30, 8815.

[7] A. S. Al‐Fatesh , R. Kumar , A. H. Fakeeha , S. O. Kasim , J. Khatri , A. A. Ibrahim , R. Arasheed , M. Alabdulsalam , M. S. Lanre , A. I. Osman , A. E. Abasaeed , A. Bagabas , Sci. Rep. 2020, 10, 13861. 32807834 10.1038/s41598-020-70930-1 PMC7431551

[8] M.‐T. Fan , K.‐P. Miao , J.‐D. Lin , H.‐B. Zhang , D.‐W. Liao , Appl. Surf. Sci. 2014, 307, 682.

[9] E. Ruckenstein , Y. H. Hu , Appl. Catal. A 1995, 133, 149.

[10] T. Borowiecki , Appl. Catal. 1987, 31, 207.

[11] S. Chen , Y. Hang Hu , Surf. Innovations 2025, 13, 84.

[12] J. Schumann , T. Lunkenbein , A. Tarasov , N. Thomas , R. Schlögl , M. Behrens , ChemCatChem 2014, 6, 2889.

[13] M. Behrens , R. Schlögl , Z. Anorg. Allg. Chem. 2013, 639, 2683.

[14] S. Zander , E. L. Kunkes , M. E. Schuster , J. Schumann , G. Weinberg , D. Teschner , N. Jacobsen , R. Schlögl , M. Behrens , Angew. Chem. 2013, 125, 6664. 10.1002/anie.201301419 23716476

[15] M. Behrens , D. Brennecke , F. Girgsdies , S. Kißner , A. Trunschke , N. Nasrudin , S. Zakaria , N. F. Idris , S. B. A. Hamid , B. Kniep , R. Fischer , W. Busser , M. Muhler , R. Schlögl , Appl. Catal. A 2011, 392, 93.

[16] P. Summa , K. Swirk , D. Wierzbicki , M. Motak , I. Alxneit , M. Rønning , P. Da Costa , Molecules 2021, 26, 6506. 34770915 10.3390/molecules26216506 PMC8588090

[17] G. Zhu , C. Xi , M. Shen , C. Bao , J. Zhu , Appl. Mater. Interfaces 2014, 6, 17208. 10.1021/am505056d 25212382

[18] J. Zheng , X. Lian , M. Wu , F. Zheng , Y. Gao , H. Niu , Diamond Rel. Mater. 2021, 116, 108451.

[19] S. Bette , C. Rincke , R. E. Dinnebier , W. Voigt , Z. Anorg. Allg. Chem. 2016, 642, 652.

[20] C. Rincke , S. Bette , R. E. Dinnebier , W. Voigt , Eur. J. Inorg. Chem. 2015, 36, 5913.

[21] S. Weber , R. T. Zimmermann , J. Bremer , K. L. Abel , D. Poppitz , N. Prinz , J. Ilsemann , S. Wendholt , Q. Yang , R. Pashminehazar , F. Monaco , P. Cloetens , X. Huang , C. Kübel , E. Kondratenko , M. Bauer , M. Bäumer , M. Zobel , R. Gläser , K. Sundmacher , T. L. Sheppard , ChemCatChem 2022, 14, e202101878.

[22] L. Zwiener , F. Girgsdies , D. Brennecke , D. Teschner , A. G. F. Machoke , R. Schlögl , E. Frei , Appl. Catal. B 2019, 249, 218.

[23] H. M. Rietveld , J. Appl. Cryst. 1969, 2, 65.

[24] M. Thommes , K. Kaneko , A. V. Neimark , J. P. Olivier , F. Rodriguez‐Reinoso , J. Rouquerol , K. S. W. Sing , Pure Appl. Chem. 2015, 87, 1051.

[25] D. Shannon , Acta Cryst. 1976, A32, 751.

[26] A. Parmaliana , F. Arena , F. Frusteri , N. Giordano , J. Chem. Soc. Faraday Trans. 1990, 86, 2663.

[27] T. H. Ulucan , J. Wang , E. Onur , S. Chen , M. Behrens , C. Weidenthaler , ACS Catal. 2024, 14, 2828. 38449535 10.1021/acscatal.3c05629 PMC10913046

[28] Y. Shimomura , Z. Nishiyama , Mem. Inst. Sci. Ind. Res. Osaka Univ. 1948, 6, 30, University.

[29] W. L. Bragg , Nature 1920, 105, 646.

[30] Q. Jeangros , T. W. Hansen , J. B. Wagner , C. D. Damsgaard , R. E. Dunin‐Borkowski , C. Hébert , J. Van herle , A. Hessler‐Wyser , J. Mater. Sci. 2013, 48, 2893.

[31] “DFG Priority Program SPP 2080 – Catalysts and reactors under dynamic conditions for energy storage and conversion”, https://www.itcp.kit.edu/spp2080/english/index.php, (accessed: 05.06.2025).

[32] S. Weber , A. Diaz , M. Holler , A. Schropp , M. Lyubomirskiy , K. L. Abel , M. Kahnt , A. Jeromin , S. Kulkarni , T. F. Keller , R. Gläser , T. L. Sheppard , Adv. Sci. 2022, 9, 2105432. 10.1002/advs.202105432 PMC8922122 35289133

[33] S. Weber , D. Batey , S. Cipiccia , M. Stehle , K. L. Abel , R. Gläser , T. L. Sheppard , Angew. Chem. Int. Ed. 2021, 60, 21772. 10.1002/anie.202106380 PMC8518723 34339595

[34] J. Huang , X. Li , X. Wang , X. Fang , H. Wang , X. Xu , J. CO2 Util. 2019, 33, 55.

[35] M. Monai , K. Jenkinson , A. E. M. Melcherts , J. N. Louwen , E. A. Irmak , S. Van Aert , T. Altantzis , C. Vogt , W. van der Stam , T. Duchon , B. Šmíd , E. Groeneveld , P. Berben , S. Bals , B. M. Weckhuysen , Science 2023, 380, 644. 37167405 10.1126/science.adf6984

[36] R. A. Van Santen , Acc. Chem. Res. 2009, 42, 57. 18986176 10.1021/ar800022m

[37] M.‐M. Millet , G. Algara‐Siller , S. Wrabetz , A. Mazheika , F. Girgsdies , D. Teschner , F. Seitz , A. Tarasov , S. V. Levchenko , R. Schlögl , E. Frei , J. Am. Chem. Soc. 2019, 141, 2451. 30640467 10.1021/jacs.8b11729 PMC6728101

[38] J. Wang , S. Chen , P. Ticali , P. Summa , S. Mai , K. Skorupska , M. Behrens , Nanoscale 2024, 16, 17378. 39189188 10.1039/d4nr02025a

[39] M.‐A. Serrer , A. Gaur , J. Jelic , S. Weber , C. Fritsch , A. H. Clark , E. Saraçi , F. Studt , J.‐D. Grunwaldt , Catal. Sci. Technol. 2020, 10, 7542.

[40] A. Coelho , J. Appl. Cryst. 2018, 51, 210.

[41] R. W. Cheary , A. A. Coelho , J. P. Cline , J. Res. Natl. Inst. Stand. Technol. 2004, 109, 1. 27366594 10.6028/jres.109.002 PMC4849620

[42] D. Balzar , N. Audebrand , M. R. Daymond , A. Fitch , A. Hewat , J. I. Langford , A. Le Bail , D. Louër , O. Masson , C. N. McCowan , N. C. Popa , P. W. Stephensj , B. H. Toby , J. Appl. Cryst. 2004, 37, 911.

[43] P. Scardi , C. L. Azanza Ricardo , C. Perez‐Demydenko , A. A. Coelho , J. Appl. Cryst. 2018, 51, 1752.

[44] M. Akao , F. Marumo , S. Iwai , Acta Crystallogr. Sect. B 1974, 30, 2670.

[45] W. J. Moore , Der Feste Zustand, Vieweg, Braunschweig 1977, pp. 99–120.

[46] G. Socrates , Infrared and Raman Characteristic Group Frequencies, John Wiley & Sons Ltd., West Sussex PO19 1UD England 2001.

[47] H. E. Swanson , E. Tatge , Circular, Vol. 539, National Bureau of Standards, USA 1953, pp. 1–95.

