# Supplementary material for: A Novel Coprecipitation Path to a High‐Performing Ni/MgO Catalyst for Carbon Dioxide Methanation
Source: ChemSusChem. 2025 Oct 9;18(23):e202502052. doi: 10.1002/cssc.202502052 (PMC12665882; doi:10.1002/cssc.202502052)
Supplement: Supplementary file 1 — Supplementary Material [file CSSC-18-e202502052-s001.pdf]

Supporting Information

Title: A novel coprecipitation path to a high performing Ni/MgO catalyst for carbon dioxide methanation

Authors: Anna Wolf,[a] Michael Chumakovski,[a] Hauke Rohr,[a] Patrik Hauberg,[a] Morteza Saedi,[a] Sebastian Mangelsen[a] and Malte Behrens*[a]

Article DOI: 10.1002/cssc.202502052

The authors have cited additional references within the Supporting Information.

Additional experimental data

For labeling the samples, the nominal values of the Ni to Mg ratio were chosen, e.g. NM9010 for a sample with a deployed relative metal atomic fractions of 90 % nickel and 10 % magnesium.

Sodium, which originates from the precipitating agent Na_2CO_3 solution, was traced with ICP-OES to cross-check an (almost) complete removal in the process of washing the precursor.

Comment on the synthesis: In principle it is possible to obtain phase pure products by replacing the automated reactor systems with manually operated traditional laboratory equipment, but the resulting products have larger particle sizes and broader size distributions as well as the frequent occurrence of by-phases due to heavily fluctuating pH and inhomogeneity during precipitation. We clearly do not recommend this approach.

Table SI-1. ICP results for all precursor compositions.

	**Ni**		**Mg**		**Na**	
Identifyer	Target value	measured value	target value	measured value	Target value	measured value
N100	100%	99.71%	0%	0.03%	0%	0.25%
NM9010	90%	89.66%	10%	9.72%	0%	0.61%
NM8020	80%	79.00%	20%	20.12%	0%	0.87%
NM7030	70%	70.32%	30%	29.05%	0%	0.62%
NM6040	60%	62.32%	40%	37.31%	0%	0.36%
NM5050	50%	56.71%	50%	42.93%	0%	0.35%
NM4060	40%	52.47%	60%	47.33%	0%	0.19%
NM3070	30%	48.67%	70%	51.17%	0%	0.15%
NM2080	20%	51.03%	80%	48.86%	0%	0.11%
NM1090	10%	42.50%	90%	57.38%	0%	0.12%

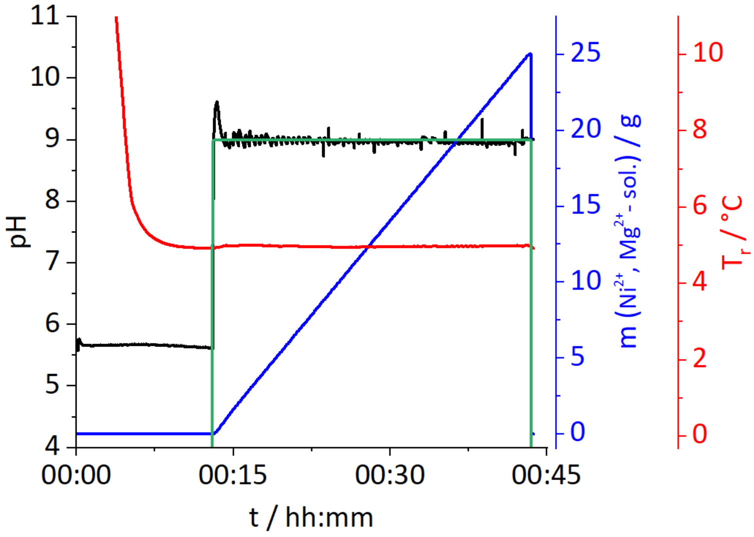

Figure SI-1. Data log of an exemplary synthesis program in the automated lab reactor system “Optimax”.

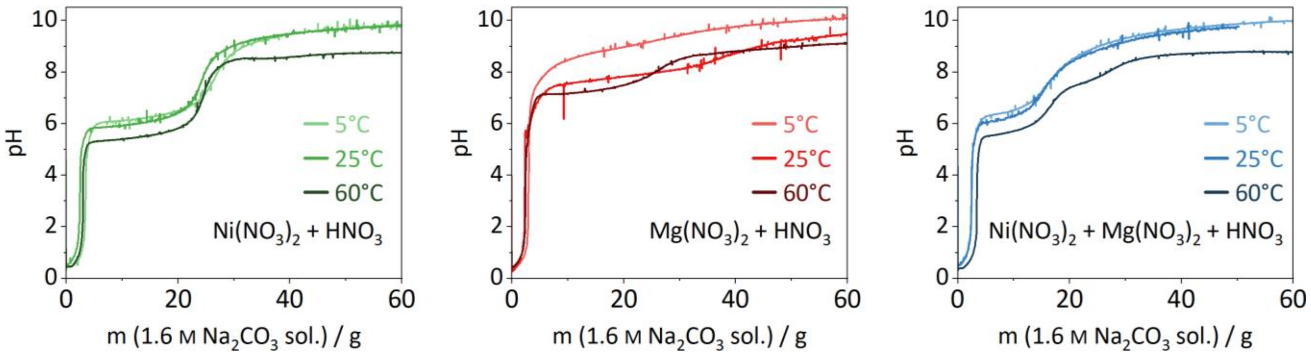

Figure SI-2. Titration experiments of pure and mixed metal nitrate solutions at different temperatures.

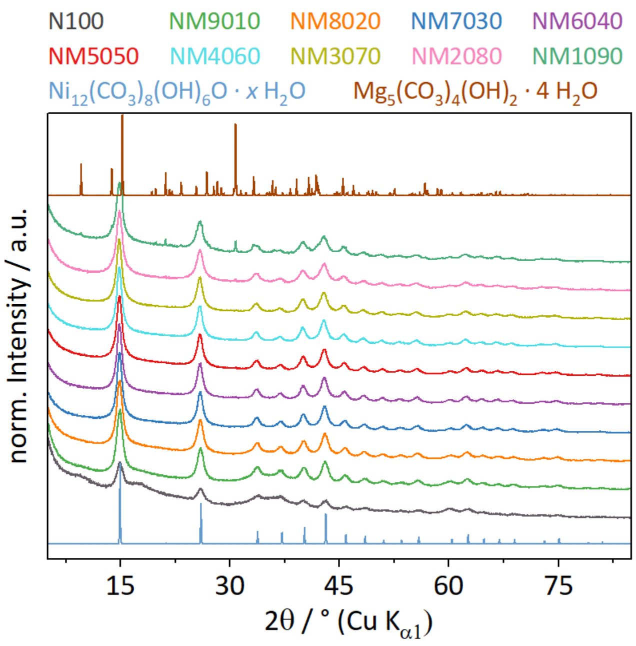

Figure SI-3. PXRD patterns of precursors with different Mg concentrations. The byphase of hydromagnesite can be identified for the samples NM4060, NM3070, NM2080 and NM1090.[21,44]

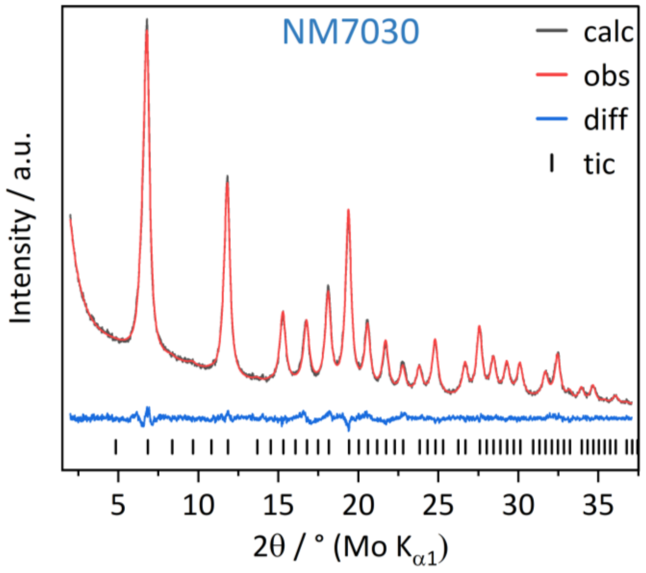

Figure SI-4. Difference plot of the Rietveld refinement of $(Ni_{0.7}Mg_{0.3})_{12}(CO_3)_8(OH)_6O$ (NM7030).

Stoichiometry

The calcination of the precursor $(Ni_{1-x}Mg_x)_{12}(CO_3)_8(OH)_6O \cdot x\ H_2O$ results in a black powder. During calcination and reduction the material undergoes several colour changes. In the literature, nickel(II)oxide is described to be of “pale apple-green” colour.[46]

The black appearance of the calcined products is not be explained with residual carbon in form of carbonate species (see chapter “calcined material”) alone, which was proven by elemental analysis. It can rather originate from the nickel oxide itself. As described by Moore[46] the crystal structure of black nickel oxide deviates from the ideal cubic NaCl structure type towards a rhombohedral structure at room temperature. Above 250 °C the ideal structure forms. The non-stoichiometry is characterized by a nickel deficiency of 2 %[46], which in this case made it experimentally not detectable. This nickel deficiency can be compensated by oxidation of some nickel atoms to Ni(III) causing the black colour.

Under reducing conditions (5% H_2 in Ar) and with a starting material of a nickel and magnesium solid solution oxide the colour changes to greenish and therefore the transition from a non stoichiometric to stoichiometric oxide could be observed at lower temperatures (210 °C) indicating a similar defect mechanism as in pure nickel oxide. When exposing the material to air the darkening of the substance occurred within minutes. Pure green stoichiometric nickel oxide as well as a solid solution with MgO which has been synthesized by calcination at high temperatures (900 °C) is on the other hand stable at ambient conditions which leads to the conclusion that the reversible colour changes at low temperatures require a non-stoichiometric starting material obtained at milder calcination.

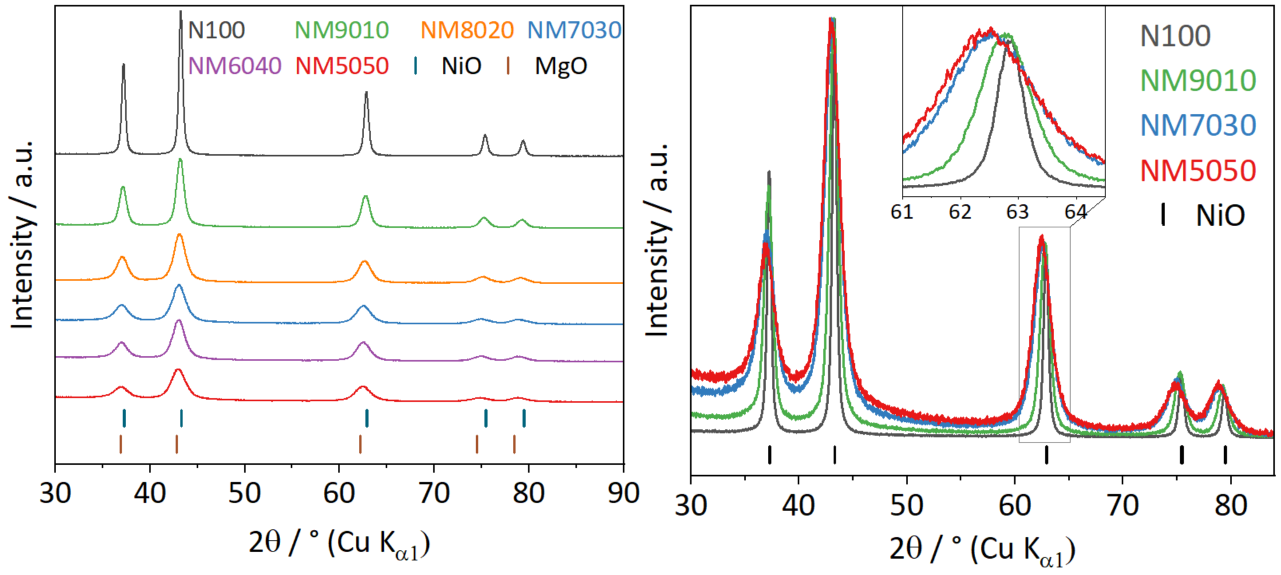

Figure SI-5. PXRD patterns of solid solution oxides with different Mg concentrations which have been calcined for 12 hours (left) and 3 hours (right).[26,27]

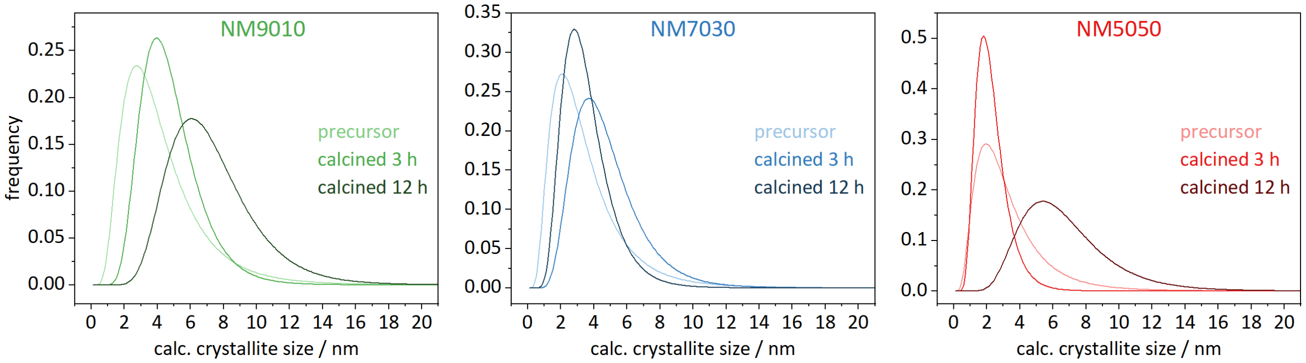

Figure SI-6. Domain sizes derived from WPPM calculations on the precursor and calcined samples of NM9010, NM7030 and NM5050.

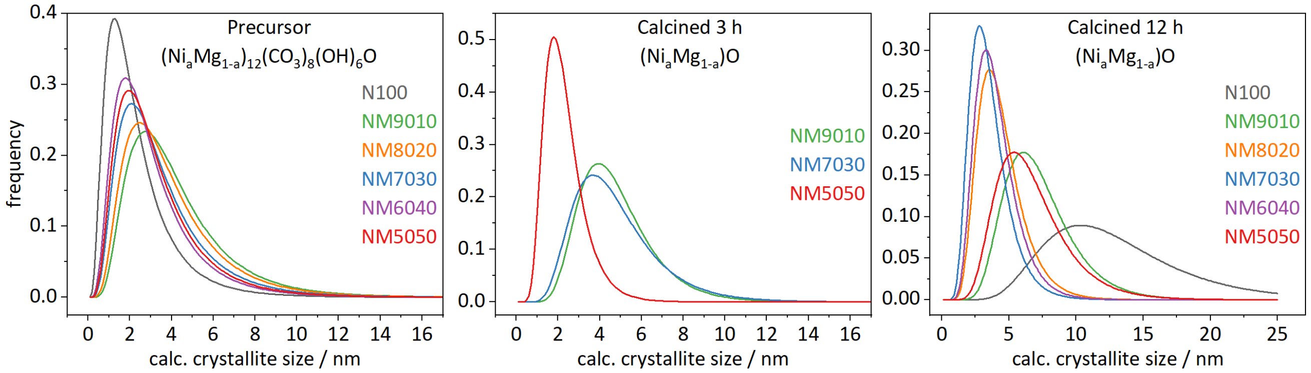

Figure SI-7. Domain sizes derived from WPPM calculations on the precursors (left) and calcined for 3 h (middle) and 12 h (right) samples of different Mg concentrations.

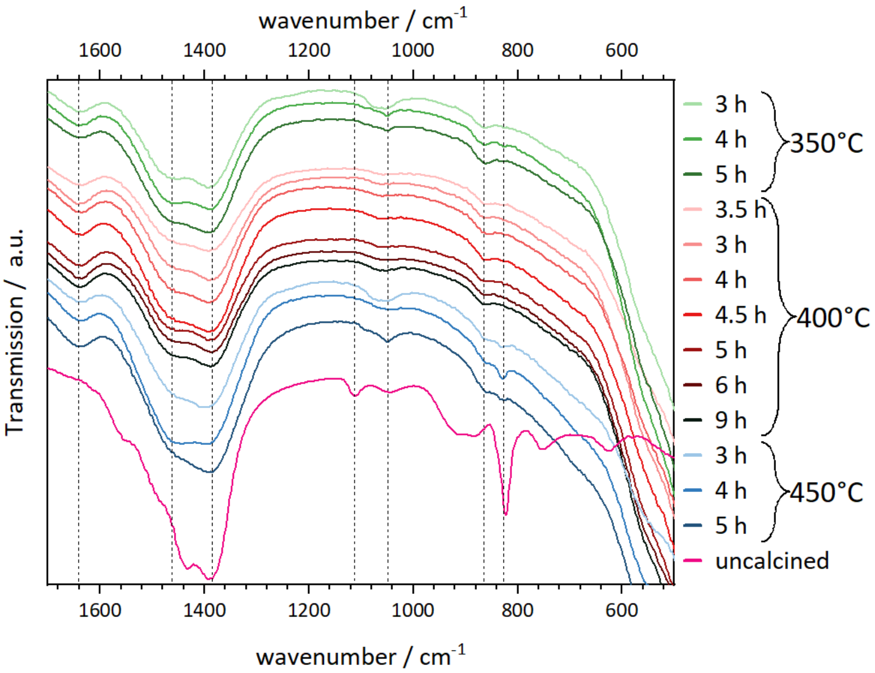

Figure SI-8. Excerpt from the IR spectra for various calcination times and temperatures as well as the uncalcined precursor (pink). It is apparent that in the materials calcined at 350 °C some bands from the precursor still occur (e.g. 828 cm^{-1}) which indicates an incomplete decomposition. The most intense band(s) from 1380-1460 cm^{-1} can be assigned to adsorbed CO_2 and water.[47]

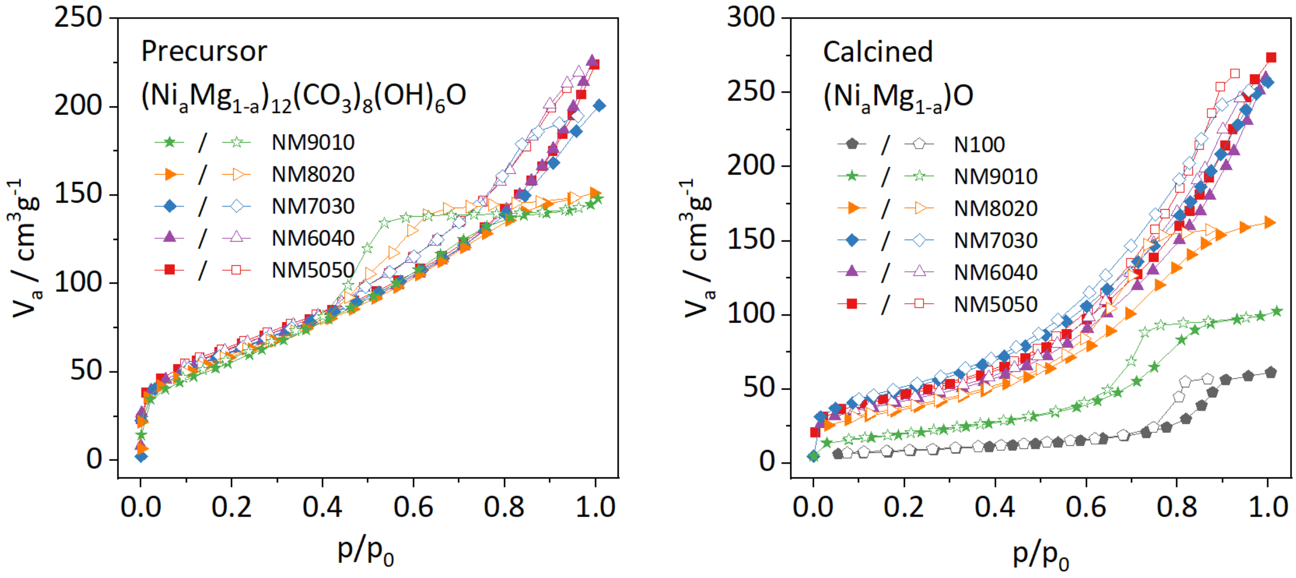

Figure SI-9. N_2-Adsorption isotherms at 77 K of precursors (left) and calcined (right) samples of different Mg concentrations.

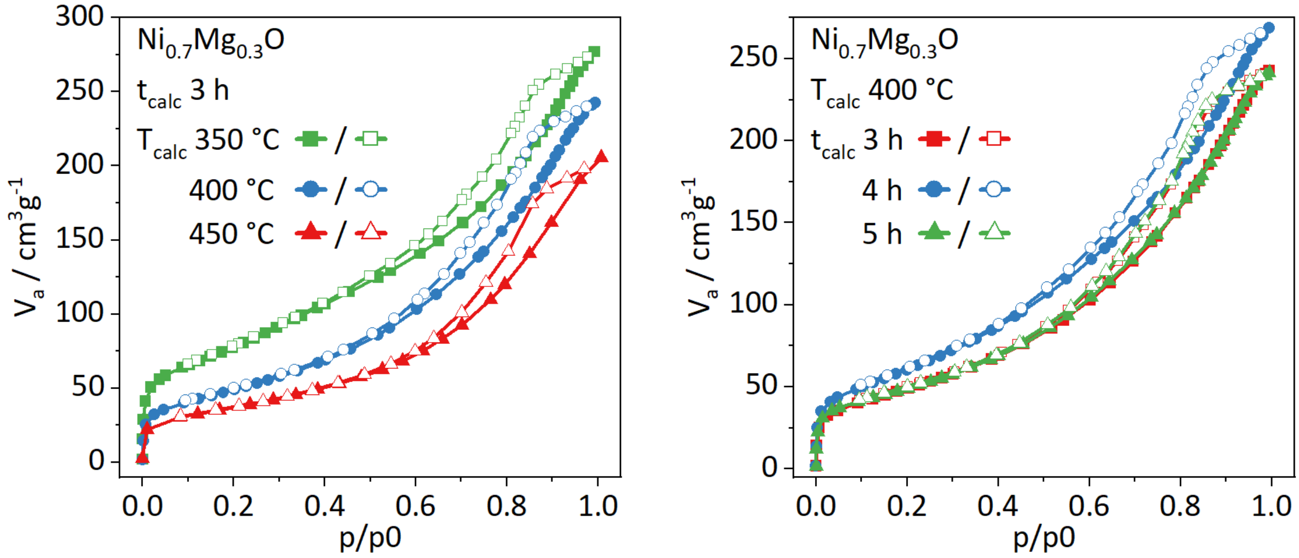

Figure SI-10. N_2-Adsorption isotherms of at 77 K calcined samples of NM7030 for different calcination temperatures and times.

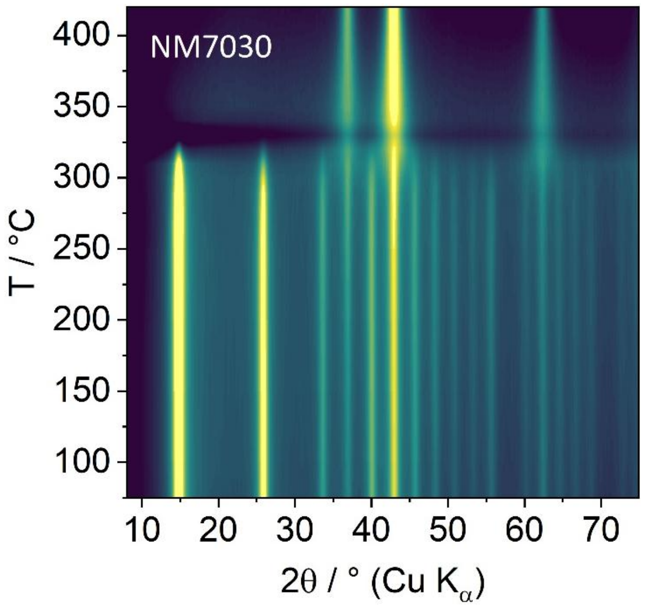

Figure SI-11. Temperature resolved PXRD experiment on a NM7030 sample in static air.

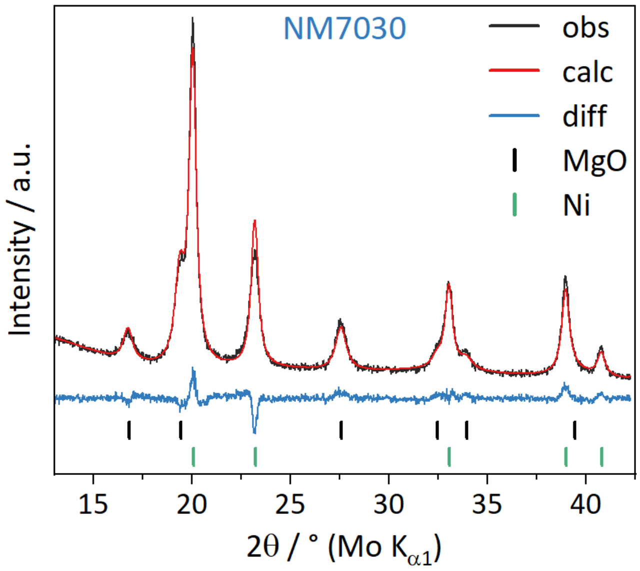

Figure SI-12. Difference plot of the Rietveld refinement of isothermally reduced NM7030 (500 °C, 3h). The fit shows metallic Ni (calculated crystallite size of approx. 6.6 nm) and cubic $Ni_xMg_{1-x}O$ (calculated crystallite sizes of approx. 4.3 nm). The refinement also shows that not all of the Ni fraction has been reduced, but that the solid solution is still partially retained, with approx. 32.2 % of the lattice sites in the periclase phase still occupied by Ni. The combined elemental composition is determined with 68.7 at.-% Ni (relative atomic percentage) which is in acceptable agreement with the ICP result of 70 %. Note also that the intensity mismatch for fcc-Ni points to stacking faults, which may also affect the phase quantification.

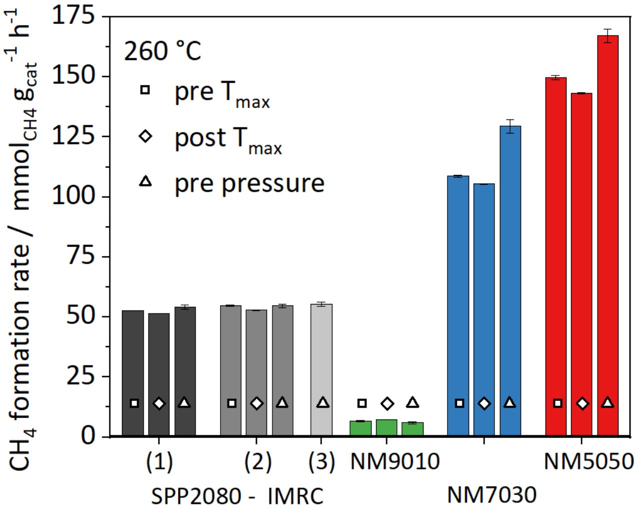

Figure SI-13. The catalysts were tested stepwise from 240 °C up to 280 °C (square: 260 °C pre T_{max}) and afterwards again at 260 °C (diamond: post T_{max}). The triangle marked measurement are the first step of the pressure increasing experiment at 0 barg. Comparing pre-T_{max} and post T_{max}, all catalysts show reasonable stability. The high thermal stability of NM9010 at low performance can likely be explained by an already largely sintered state after reduction due to its high nickel content. The higher formation rate from the pressure test experiment can be assigned to a shorter time on stream (TOS) which is in this case max. 2.7 h and for the temperature variation experiments 10 – 13 h (pre T_{max}) and 26 – 29 h (post T_{max}) TOS.

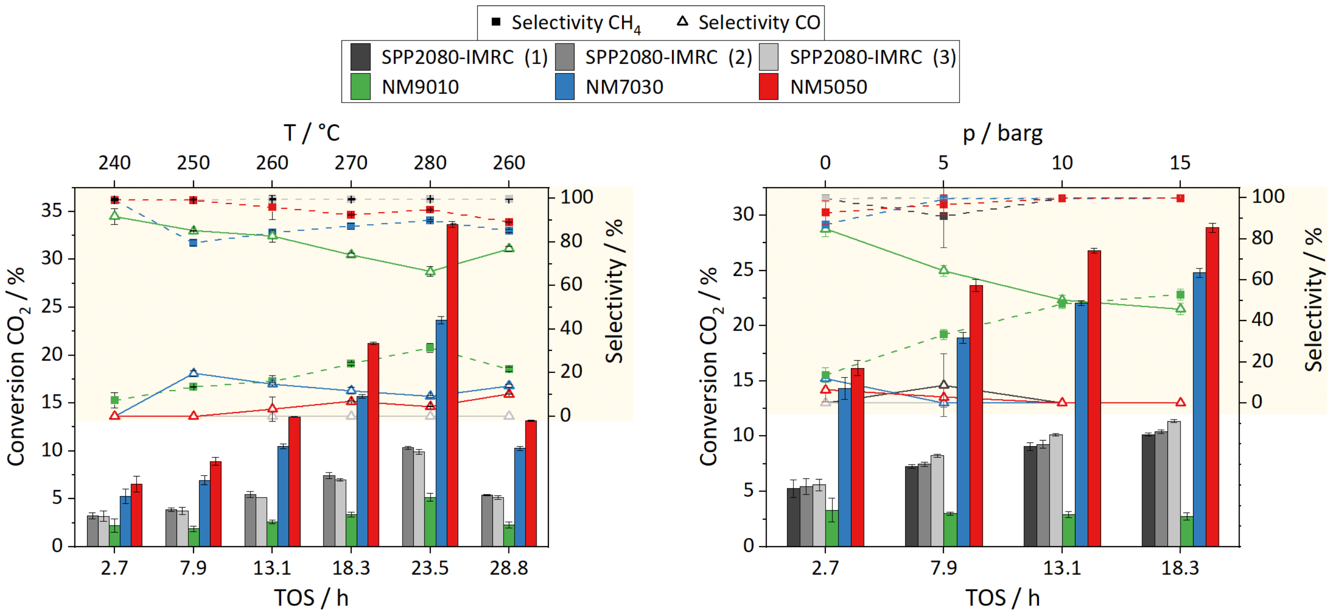

Figure SI-14. Conversion of CO_2 and selectivity towards methane (CH_4) or carbon monoxide (CO).

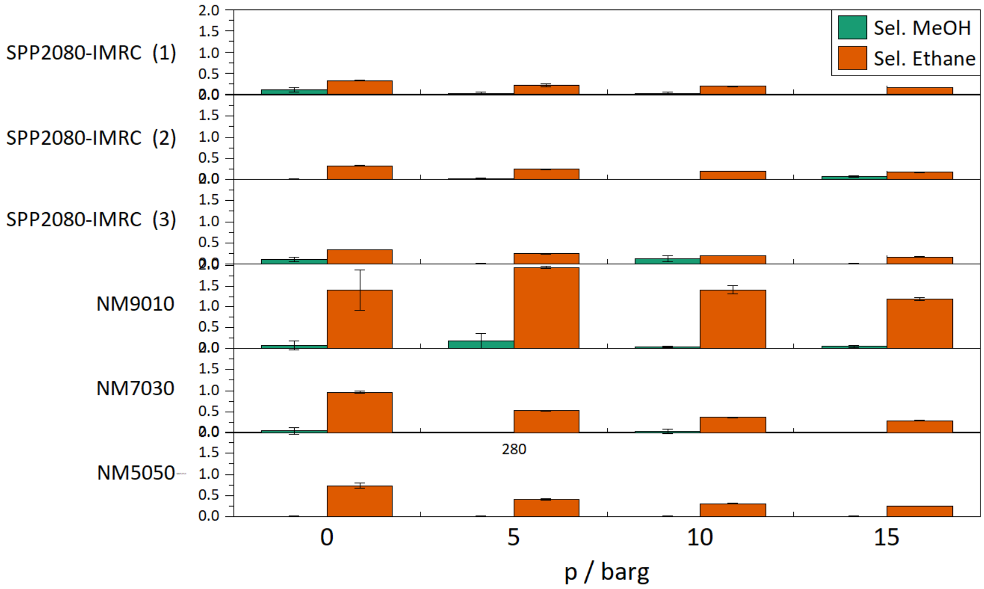

Figure SI-15. Selectivity (%) towards traces of ethane (C_2H_6) and methanol (CH_3OH) for different pressures.

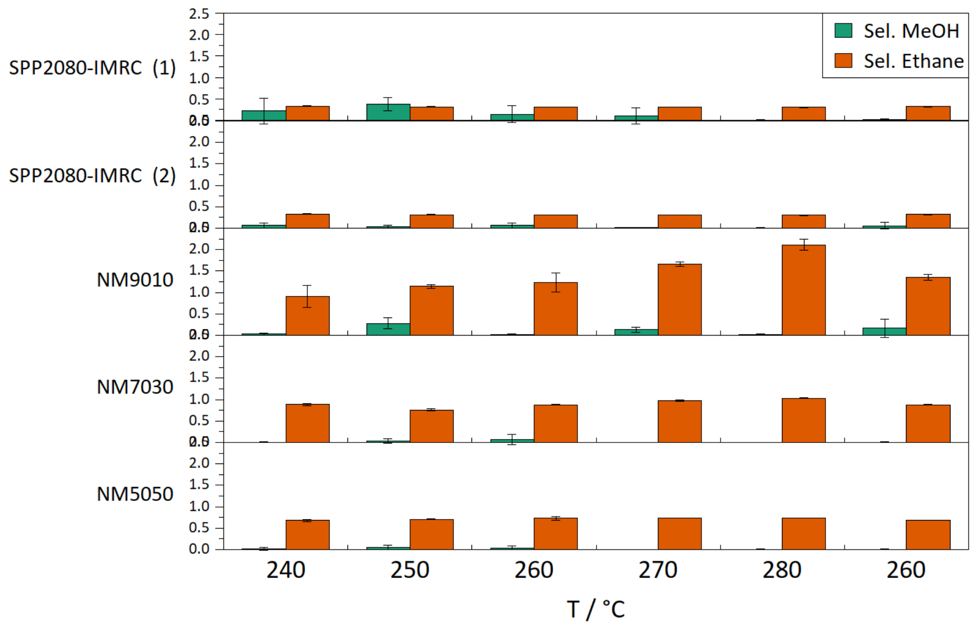

Figure SI-16. Selectivity (%) towards traces of ethane (C_2H_6) and methanol (CH_3OH) for different temperatures.

Table SI-2. Carbon balance values (%) for catalytic temperature variation experiments at 0 barg (=1atm).

T / °C	SPP2080-IMRC (1)	Er ±	NM7030	Er ±	NM5050	Er ±	SPP2080-IMRC (2)	Er ±	NM9010	Er ±
**240**	99,56	0,29	99,00	0,66	99,18	0,77	99,53	0,52	100,34	0,56
**250**	100,09	0,21	101,19	0,58	100,04	0,39	100,07	0,39	100,82	0,33
**260**	100,09	0,29	101,46	0,29	101,03	0,86	100,26	0,01	101,57	0,92
**270**	100,29	0,34	102,20	0,51	102,68	0,21	100,51	0,15	101,55	0,42
**280**	100,29	0,18	102,86	0,35	104,31	0,12	100,88	1,07	101,20	0,15
**260**	100,01	0,08	101,19	0,25	101,86	0,04	100,07	0,19	100,95	0,19

Table SI-3. Carbon balance values (%) for catalytic pressure variation experiments at 260 °C.

p / barg	SPP 2080-IMRC (1)	Er ±	NM 7030	Er ±	SPP 2080-IMRC (2)	Er ±	NM 9010	Er ±	SPP 2080-IMRC (3)	Er ±	NM 5050	Er ±
**0**	99,70	0,80	101,47	0,86	99,42	0,74	101,10	2,36	99,79	0,53	101,06	1,62
**5**	101,84	1,75	101,67	0,31	100,63	0,21	100,63	0,21	100,61	0,12	103,13	1,38
**10**	100,76	0,33	102,21	0,31	100,63	0,35	100,68	0,37	100,92	0,05	103,11	0,04
**15**	100,88	0,10	102,47	0,37	100,83	0,19	100,71	0,49	101,11	0,13	103,42	0,20

Table SI-4. Weight of undiluted catalysts prior to dilution with SiC (50-55 mg) and loading in the reactor.

	**Catalyst**	**mass / mg**		**Catalyst**	**mass / mg**
**T variation**	SPP2080-IMRC (1)	11.7	**p variation**	SPP2080-IMRC (1)	10.4
	NM7030	10.3		NM7030	12.0
	NM5050	10.4		SPP2080-IMRC (2)	10.1
	SPP2080-IMRC (2)	11.0		NM9010	10.5
	NM9010	10.8		SPP2080-IMRC (3)	11.0
				NM5050	10.8
